# IGF2BP2 binding to CPSF6 facilitates m6A‐mediated alternative polyadenylation of PUM2 and promotes malignant progression in ovarian cancer

**DOI:** 10.1002/ctm2.70388

**Published:** 2025-07-09

**Authors:** Xin Luo, Qinglv Wei, Lingcui Xie, Ningxuan Chen, Bin Gu, Jiani Xu, Xiaoyan Jiang, Xinzhao Zuo, Hongyan Zhao, Xiaoyi Liu, Yu Yang, Tao Liu, Yong Zhu, Ping Yi, Jing Xu

**Affiliations:** ^1^ Department of Obstetrics and Gynecology The Third Affiliated Hospital of Chongqing Medical University Chongqing P.R. China; ^2^ Chongqing Municipal Health Commission Key Laboratory of Basic and Clinical Transformation of Gynecological Oncology The Third Affiliated Hospital of Chongqing Medical University Chongqing P.R. China; ^3^ Chongqing Key Laboratory of Child Infection and Immunity Children's Hospital of Chongqing Medical University Chongqing P.R. China; ^4^ School of Basic Medicine Chongqing Medical University Chongqing P.R. China; ^5^ School of Basic Medicine Hubei University of Medicine Shiyan P.R. China

**Keywords:** alternative polyadenylation, CPSF6, IGF2BP2, N6‐methyladenosine, ovarian cancer, PUM2

## Abstract

**Background:**

N6‐methyladenosine (m6A) and alternative polyadenylation (APA) are common posttranscriptional regulatory mechanisms in eukaryotes. However, the m6A‐dependent mechanism of APA regulation in ovarian cancer (OC) is still unclear.

**Methods:**

The correlation between m6A and APA was analyzed by using RNA methylation sequencing of OC cells and single‐cell sequencing of clinical samples from public databases. To explore the core regulatory factors that served as a bridge between m6A and APA, we employed RNA pull‐down with biotin‐labelled m6A, immunoprecipitation, mass spectrometry, western blot, protein purification and GST pull‐down assays. Furthermore, the important target genes were screened by PAS‐seq, eCLIP‐seq, RIP‐seq and meRIP‐seq, and verified by RT‐qPCR, 3′RACE, RNA stability, and dual luciferase reporter assays. Multiple phenotypic experiments were conducted to evaluate the function of the IGF2BP2‐PUM2 axis in vitro and in vivo.

**Results:**

This study found that the m6A was correlated with the APA and affected the 3′end processing in OC. The APA regulator CPSF6 tended to bind the m6A‐modified transcripts in OC cells. Mechanistically, we demonstrated that the m6A reader IGF2BP2 KH1‐4 domains could directly bind to the CPSF6‐RS domain to regulate the 3′end processing of OC. Furthermore, sequencing revealed that the m6A was highly enriched in the 3′UTR near the proximal polyadenylation signal (PAS), which promotes the use of proximal PAS and leads to 3′UTR shortening. PUM2 was carried m6A and recognized by IGF2BP2, and CPSF6 was recruited at the proximal polyadenylation signal (pPAS) to generate the short‐3′UTR transcript. The short PUM2 transcript was more stable than the long transcript, which promoted the malignant progression of OC.

**Conclusions:**

We revealed a novel mechanism in which the m6A could regulate the APA processing of pre‐mRNAs by crosstalk of IGF2BP2 and CPSF6. This study provides a potential strategy for the effective treatment of OC.

**Highlights:**

- The interaction between m6A and APA is mediated by the m6A regulator IGF2BP2 and the APA factor CPSF6.
- The transcripts harboring m6A modification tend to use the proximal polyadenylation signal (PAS) in ovarian cancer (OC).
- PUM2 promotes the malignant progression of OC through its m6A methylation and APA processing.

## Graphical abstract

1. The interaction between m6A and APA is mediated by the m6A regulator IGF2BP2 and the APA factor CPSF6
2. The transcripts harbouring m6A modification tend to use the proximal polyadenylation signal (PAS) in ovarian cancer (OC)
3. PUM2 promotes the malignant progression of OC through its m6A methylation and APA processing

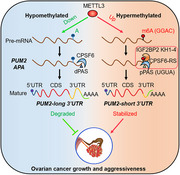

## 1 INTRODUCTION

Ovarian cancer (OC) ranks first in mortality among all gynecologic malignancies.^1^ The ratio of recurrence or metastasis in OC patients is approximately 70% within 3 years, with only a 30% 5‐year survival rate for advanced OC.^2^ Thus, it is crucial to investigate novel mechanisms and targets aimed at improving the prognosis of OC individuals.

N6‐methyladenosine (m6A) is the most abundant internal modification in eukaryotic cells.^3^ This chemical mark is linked to various facets of RNA metabolism, including stability, alternative exon usage, nuclear export, degradation, and protein synthesis, which mediated multiple pathological processes and disease states, including carcinogenesis.^3^ Recent studies frequently demonstrated that m6A regulator acted as oncogene to promote the occurrence, development and chemotherapy resistance through regulating m6A modification in OC, including methyltransferase METTL3/METTL14/WTAP, demethylase ALKBH5, recognized proteins YTHDC2, YTHDF1/2, and IGF2BP1/2/3.^4^, ^5^, ^6^, ^7^, ^8^, ^9^, ^10^, ^11^, ^12^, ^13^, ^14^, ^15^, ^16^, ^17^, ^18^ In our laboratory, we discovered that YTHDF1 promoted OC progression by interacting with EIF3C mRNA and boosting overall translation through an m6A‐dependent manner.^19^ Recently, m6A modification was found to regulate the alternative cleavage and polyadenylation (APA) in OC.^20^

APA is a widely conserved method of regulating gene expression after transcription.^21^ Over 70% of mammalian mRNAs possess multiple polyadenylation sites, leading to diverse transcriptomes and proteomes.^22^ In humans, the processing of pre‐mRNA at the 3′ end is facilitated by the cleavage and polyadenylation (CPA) complex, orchestrated by four protein subcomplexes: cleavage stimulation factors (CSTFs), cleavage and polyadenylation specific factors (CPSFs), cleavage factor I mammalian (CFIm) and CFIIm, along with numerous subunits.^23^ Our group has reported that APA factor CSTF3 facilitated OC platinum resistance by modulating the 3′end processing of NEAT1, favouring the production of its shorter isoform, NEAT1_1, and we are delving into the unknown areas of APA regulation.^24^

In this study, we observed that m6A could affect pre‐mRNA 3′end processing, in which the IGF2BP2 KH1‐4 region directly interacts with the CPSF6 RS domain to facilitate pPAS usage in methylated transcripts. Knockdown of METTL3 or IGF2BP2 affected the APA profiles of numerous genes, and the usage of PAS switched from proximal to distal. Furthermore, we verified a METTL3/IGF2BP2/CPSF6‐modulated APA event in PUM2 pre‐mRNA to enhance the mRNA stability and promote malignant progression of OC. These findings reveal a novel mechanism involving the crosstalk of m6A and APA, which may provide a promising strategy for OC therapy.

## 2 MATERIALS AND METHODS

### 2.1 Clinical tissue sample collection

Twelve paired OC tissue samples (histologically confirmed high‐grade serous carcinoma) and normal fallopian tube epithelial tissues were collected from treatment‐naive patients undergoing primary cytoreductive surgery at Third Affiliated Hospital of Chongqing Medical University. All specimens were conducted under the ethical guidelines sanctioned by the Institutional Review Board of the Third Affiliated Hospital of Chongqing Medical University (approval no. 202293) in accordance with the Declaration of Helsinki. All participants provided written informed consent before undergoing surgery. Tumour tissues were collected from the ovarian neoplasms immediately after surgical resection, while normal controls were harvested from the fimbriated end of contralateral fallopian tubes showing no pathological abnormalities upon intraoperative frozen section evaluation.

### 2.2 Cell culture and transfection

The Laboratory of Obstetrics and Gynecology at the Clinical Research Center of the Third Affiliated Hospital of Chongqing Medical University provided A2780, OVCAR3, SKOV3, HeLa, and HEK 293T (293T) cells. A2780, OVCAR3 and SKOV3 were grown in RPMI 1640 (Gibco), and HeLa and 293T cells were cultured using DMEM (Gibco) supplemented with 10% FBS (Biological Industries) and 1% penicillin/streptomycin (Sigma). All cells were cultured in fresh medium at 37°C in a humidified incubator with 5% CO_2_. Cells were transfected with plasmids using jetPRIME reagent (Polyplus Transfection).

### 2.3 Plasmid construction, lentiviral infection, and inhibitor treatment

To generate lentiviral shRNA constructs, sequence‐specific forward and reverse oligonucleotides targeting METTL3, IGF2BP2, and PUM2 transcripts were commercially synthesized by Tsingke Biotechnology Co., Ltd. Following restriction enzyme digestion (AgeI/EcoRI), the annealed duplexes were directionally subcloned into the commercially sourced pLKO.1 vector (Addgene). Comprehensive shRNA sequence information is documented in Table S1. For viral particle production, HEK293T cells were co‐transfected with the shRNA plasmids alongside psPAX2 packaging plasmid and pMD2.G envelope plasmid. Culture supernatants containing viral particles were harvested 48–72 h after and subsequently applied to infect target cells over a 24 h incubation period. For overexpressing genes, the pcDNA3.1‐CPSF6‐FL‐FLAG, pcDNA3.1‐CPSF6‐ΔRRM‐FLAG, pcDNA3.1‐CPSF6‐ΔPRR‐FLAG, pcDNA3.1‐CPSF6‐ΔRS‐FLAG, pcDNA3.0‐IGF2BP1‐MYC, pcDNA3.1‐IGF2BP2‐MYC, pcDNA3.1‐IGF2BP3‐MYC, pcDNA3.1‐IGF2BP2‐RRM‐MYC, pcDNA3.1‐IGF2BP2‐KH‐1‐4‐MYC, pcDNA3.1‐IGF2BP2‐KH1‐2‐MYC, and pcDNA3.1‐IGF2BP2‐KH3‐4‐MYC plasmids were produced by Tsing Ke. The CPSF6 plasmid (pENTER‐CPSF6‐FLAG) was acquired from WZ Biosciences. PUM2 3′UTR wild‐type (WT)/mutant (MUT) and pcDNA3.1‐PUM2‐FLAG plasmids were constructed by PAIVIBIO Company. All of the corresponding empty vector plasmids acted as negative controls. For inhibiting molecular function, the cells were treated with METTL3 inhibitor STM2457 (10 µM) and IGF2BP2 inhibitor CWI1‐2 (5 µM) for 48 h.

### 2.4 Western blot

Cells and tissues were subjected to protein extraction with RIPA lysis buffer (Beyotime) supplemented with 1% PMSF, and the concentrations were assessed using a BCA protein assay kit (Solarbio). Equivalent amounts of protein (20 µg per lane) were loaded onto 10% SDS‐PAGE gels for electrophoretic separation, followed by transfer to PVDF membranes (Millipore). The membranes were blocked in 5% skim milk at room temperature for 1 h and then incubated with primary antibodies overnight at 4°C. Immunological assays utilized commercially available antibodies targeting key molecular complexes: core cleavage/polyadenylation machinery components included CPSF6 (Bethyl A301‐358A), PCF11 (A303‐705A), Symplekin (A301‐465A), CPSF1‐3 (A301‐580A/A301‐581A/A301‐091A), FIP1 (A301‐462A), CSTF1‐3 (A301‐250A/A301‐092A/A301‐095A), CPSF7 (Bethyl A301‐359A), and NUDT21 (Proteintech 10322‐1‐AP). The m6A regulatory axis was probed using VIRMA (Proteintech 25712‐1‐AP), METTL3/14 (15073‐1‐AP/26158‐1‐AP), WTAP (60188‐1‐Ig), FTO (27226‐1‐AP), and ALKBH5 (16837‐1‐AP). RNA‐binding proteins were detected with YTHDC1 (Abcam ab220159), HnRNPC/A2B1 (Proteintech 11760‐1‐AP/14813‐1‐AP), YTHDF1/2 (17479‐1‐AP/24744‐1‐AP), and IGF2BP1/2/3 (22803‐1‐AP/11601‐1‐AP/14642‐1‐AP). Epitope tags were verified using FLAG (MBL M185‐3L), MYC (M192‐3), His (D291‐3), and GST (M209‐3) antibodies. The PUM2 (Abcam, ab92390) and SNRNP70 (Abcam, ab316762) were used, with Tubulin (Proteintech 11224‐1‐AP) and GAPDH (60004‐1‐Ig) serving as loading controls. The following day, the membrane was treated with the appropriate HRP‐linked secondary antibody for 1 h at ambient temperature, and then the proteins were identified using enhanced chemiluminescence. The expression was measured by analyzing the band density and adjusted according to the GAPDH level.

### 2.5 RNA extraction and RT‒qPCR

Total RNA was isolated from transfected cells using TRIzol Reagent (Invitrogen) according to the manufacturer's protocol. HiScript III RT SuperMix (Vazyme) was used to synthesize first‐strand cDNA from 1 µg of total RNA under these conditions: 50°C for 15 min, 85°C for 5 s. Quantitative PCR amplification was conducted on an ABI 7500 Real‐Time PCR system with ChamQ Universal SYBR qPCR Master Mix (Vazyme, Q711‐02). Custom‐designed primers (Tsingke Biotech, sequences in Table S1) were validated for amplification efficiency (90–110%) and specificity (single‐peak melting curves). Relative gene expression levels were quantified using the 2^−ΔΔCt^ method with GAPDH as the endogenous reference.

### 2.6 RNA‒protein pulldown assay

Biotin‐labelled RNA oligonucleotides corresponding to the same sequence—ss‐A (unmethylated control) and ss‐m6A (bearing the GG(m6A)CU consensus motif)—were synthesized by GE Dharmacon as previously reported.^25^ Briefly, 1.5 µL of RNA probes (100 pM final concentration) was heated at 99°C for 10 min for denaturation, followed by rapid cooling on ice. The denatured probes were incubated with 50 µL of washed dynabeads protein G (Invitrogen, 10004D) with RNA capture buffer (20 mM Tris‐HCl pH 7.5, 1 M NaCl, 50 mM KCl, 1 mM EDTA, 1 × protease and phosphatase inhibitor cocktail) at 4°C for 4 h and then incubated with cell protein lysates in RNA‒protein binding buffer containing 200 mM Tris‐HCl (pH 7.5), 500 mM NaCl, 20 mM MgCl₂, and 1% Tween‐20 at 4°C overnight. RNA–protein complexes associated with magnetic beads were separated from unbound materials. After denaturation and elution, the pulled‐down proteins were detected by western blotting.

### 2.7 Coimmunoprecipitation and mass spectrometry

Cellular lysates were prepared using ice‐cold IP buffer composed of 20 mM HEPES (pH 7.4), 150 mM NaCl, 10 mM KCl, 3.75 mM MgCl, 0.5 mM EDTA, 10% glycerol, and 0.5% NP‐40, freshly supplemented with 1 mM DTT and a 1 × concentration of protease inhibitor cocktail (PIC). A 5% aliquot of the total lysate was reserved as an input control. Clarified supernatants were subjected to immunoprecipitation through overnight rotation (4°C) with 3 µg target‐specific antibodies or species‐matched IgG controls, followed by 3 h incubation with 50 µL pre‐equilibrated Protein G magnetic beads (Invitrogen, 10004D). Bead complexes were magnetically captured and sequentially washed five times with lysis buffer before resuspension in 2 × SDS buffer. For immunoblot validation, samples were heat‐denatured (75°C, 10 min), resolved on 10% SDS‐polyacrylamide gels, and electrotransferred to PVDF membranes. Parallel mass spectrometry (MS) samples were processed identically except for RNase A treatment (200 ng/mL; Invitrogen EN0531) during lysis. CPSF6 immunocomplexes were specifically enriched using a validated rabbit polyclonal antibody (Proteintech 15489‐1‐AP), with rabbit IgG (Millipore 12–370) serving as a negative control. Finally, the proteins were detected by silver and subjected to MS analysis by BGI (BGI‐Shenzhen).

### 2.8 Computational prediction of protein–protein interaction

The structural interaction between the RS domain of CPSF6 (residues 481–551, UniProt ID: Q16630) and the KH domains of IGF2BP2 (UniProt ID: Q9Y6M1) was predicted using AlphaFold3. The top‐ranked model was selected based on confidence metrics: pLDDT > 85 at the interface, pTM > 0.8 for global accuracy, and PAE < 6 Å for interdomain alignment precision. Then, structural visualization analysis was performed in PyMOL Molecular Graphics System. And interface analysis using PDBePISA identified hydrogen bonds (distance ≤3.5 Å, donor‐H‐acceptor angle ≥120°) and salt bridges (oppositely charged residues within 4.0 Å).

### 2.9 Hematoxylin–eosin and immunohistochemistry staining

The tissue samples were fixed with 4% paraformaldehyde and subsequently sent to Servicebio Biotechnology Co., Ltd. The company was entrusted to perform hematoxylin–eosin (HE) staining and immunohistochemical (IHC) staining. IHC analysis was conducted to detect the expression levels of Ki‐67 and Caspase‐3. All sections were observed and photographed under a microscope, and the staining results were statistically analyzed using Image‐Pro Plus software.

### 2.10 Nuclear and cytoplasmic protein extraction

A nuclear and cytoplasmic extraction kit (Beyotime, P0028) was used for nuclear and cytoplasmic separation of OC cells. Briefly, fresh cells were collected and washed two times with ice‐cold PBS. The cells were lysed in extraction lysis buffer and incubated for 15 min at 4°C. The lysate was centrifuged at 1000×*g* for 10 min at 4°C, and the supernatant was carefully transferred to a new microcentrifuge tube. This fraction primarily contained cytoplasmic proteins. Subsequently, the remaining pellet was resuspended in RIPA buffer and lysed on ice for 30 min. The lysis solution was centrifuged at 12 000×*g* at 4°C for 10 min, and the supernatant was collected as the nuclear fraction. Finally, all the proteins were quantified and tested by western blotting.

### 2.11 Immunofluorescence staining

Cultured cells were plated in 24‐well plates and processed through sequential fixation/permeabilization steps: initial fixation in 4% paraformaldehyde (PFA) solution (15 min), followed by membrane permeabilization with 0.2% Triton X‐100 (10 min). Nonspecific binding sites were blocked using 1% bovine serum albumin (BSA) for 60 min at room temperature. Target antigens were probed with primary antibodies against CPSF6 (Santa Cruz Biotechnology, sc‐100692) and IGF2BP family members (Proteintech: IGF2BP1 22803‐1‐AP; IGF2BP2 11601‐1‐AP; IGF2BP3 14642‐1‐AP) through overnight incubation at 4°C. Following three PBS washes (5 min each), antigen–antibody complexes were visualized using species‐matched secondary reagents: Alexa Fluor 488‐conjugated goat anti‐rabbit IgG (Abcam ab150077) and Alexa Fluor 647‐conjugated goat anti‐mouse IgG (ab150115), applied for 60 min under light‐protected conditions. Nuclei were counterstained with DAPI (ab104139). Fluorescent signals were captured using a confocal microscope with appropriate filter sets.

### 2.12 In vitro GST pulldown assay

To obtain the GST‐CPSF6‐RS, His‐IGF2BP1, His‐IGF2BP2, and His‐IGF2BP3 proteins from *E. coli*, the CPSF6‐RS protein was cloned and inserted into multiple cloning sites in pGEX‐6P‐1 and purified using glutathione sepharose 4B MEDION (GE, 17‐0756‐04), PMAL‐C2X was used to produce 6xHis‐IGF2BP1/2/3 in *E. coli*, and the proteins were purified using Complete HIS‐Tag purification resin (ROCHE, 05893801001) per the manufacturer's instructions. Pulldown assays were performed with the GST‐fusion protein CPSF6‐RS as a probe protein to pull down His‐IGF2BP1/2/3 using GST‐tag purification resin (Beyotime, P2251) in vitro. Briefly, the resin was equilibrated with binding buffer and incubated with 100 µL of 50 µg of purified GST‐CPSF6‐RS. After 2 h at 4°C, the resin was washed two times, then 50 µg of the IGF2BP1/2/3 protein mixture was added and incubated overnight at 4°C. The binding resin was washed three times and boiled for 5 min with 2 × SDS‒PAGE loading buffer to elute proteins. All eluted samples and inputs were verified by western blotting.

### 2.13 RNA‐seq

Total RNA was isolated from OVCAR3 cells with METTL3 or IGF2BP2 knockdown using TRIzol Reagent. The RNA integrity was tested using an Agilent 2100 Bioanalyzer (RIN > 8.0). RNA‐seq libraries specific to strands were created through the NEBNext Ultra II Directional RNA Library Prep Kit and sequenced on Illumina NovaSeq 6000 by Novogene Corporation. Raw reads were quality‐controlled using FastQC and processed with Trimmomatic to remove adapters and low‐quality bases. Clean reads aligned with the human reference genome (GRCh38.p13) using HISAT2, and transcript quantification performed via featureCounts. Differential expression analysis was conducted in R using DESeq2 under default parameters. Genes with |log2(fold change)|>0.585 (FC > 1.5) and adjusted *p*‐value < 0.05 were defined as differentially expressed. Functional enrichment analysis was performed using clusterProfiler with Gene Ontology (GO) and KEGG databases.

### 2.14 PAS‐seq

Following confirmation of target gene knockdown efficiency and mycoplasma‐free validation in transduced cells, strand‐specific RNA libraries were constructed using the QuantSeq 3′ mRNA‐Seq Library Prep Kit (Lexogen, 016.24) through sequential enzymatic steps: initial cDNA synthesis through oligo(dT) primers comprising Illumina adapter sequences, followed by second‐strand generation using random hexamers with complementary linkers. Double‐stranded DNA libraries underwent magnetic bead‐based size selection and PCR amplification (15 cycles) with index‐incorporated adapters for multiplex sequencing. Final library as detected on Illumina NovaSeq 6000 platform at Annoroad Gene Technology. Bioinformatics processing involved Bowtie2 alignment against GRCh38.p5 and PolyA‐miner analysis with entropy‐based PAS detection, identifying alternative polyadenylation events using stringent thresholds as previously established.^26^

### 2.15 RIP‐Seq

RNA immunoprecipitation (RIP) was performed as described above. Cells were lysed in IP buffer containing 1 mM DTT, 1 × PIC, and 40 U/µL RNase inhibitor (Invitrogen, EO0382), and 5% (v/v) of the clarified lysate was aliquoted as input control for subsequent analysis. Anti‐IGF2BP2 antibody (Proteintech, 11601‐1‐AP) or rabbit IgG was added to the lysates for RIP at 4°C overnight. Following overnight IP, pre‐equilibrated 50 µL Protein G magnetic beads (Invitrogen 10004D) were introduced to the antigen–antibody complexes and rotated at 4°C for 4 h. Bead‐bound complexes underwent three washes with IP buffer, with 10% of each sample aliquoted for immunoblot validation. Co‐precipitated RNAs were isolated through TRIzol‐chloroform separation (Invitrogen) followed by glycogen‐facilitated isopropanol precipitation. Ribosomal RNA depletion was achieved using the NEBNext rRNA depletion kit (New England BioLabs). Strand‐specific RNA‐seq libraries were constructed and sequenced by Novogene Corporation on an Illumina NovaSeq 6000 platform. Bioinformatics analysis in R identified differentially enriched RNAs via DESeq2 with stringent thresholds (adjusted *p* < 0.05, |log2FC|>1.5).

### 2.16 MeRIP‐Seq

OVCAR3 cells underwent m6A‐specific RNA immunoprecipitation (meRIP) using magnetic bead‐conjugated anti‐m6A antibodies in optimized IP buffer through rotational incubation (4°C, 4 h). Postimmunoprecipitation, input and m6A‐enriched RNA complexes were reverse‐transcribed using SuperScript II reverse transcriptase (Invitrogen 1896649), followed by cDNA purification via AMPure XP bead selection. Uracil‐containing second‐strand DNA products were treated with UDG enzyme (NEB M0280, 37°C/30 min) to eliminate deamination artefacts prior to adapter ligation. Final library amplification employed 12‐cycle PCR with indexed primers, followed by size selection (200–500 bp). Paired‐end sequencing (150 bp) was conducted by LC‐Bio Technology on an Illumina NovaSeq platform. Data analysis was performed in the Python and R programming environments. Especially, we performed an integrative analysis of m6A‐seq and single‐cell RNA‐seq (scRNA‐seq) data from OC tissue samples (GSE154600). m6A‐modified transcripts were identified using MACS2 peak calling with anti‐m6A antibody‐enriched regions, while APA dynamics were quantified from scRNA‐seq data using Sierra to detect transcript terminal sites (TTS). Gene‐level overlaps between m6A‐modified genes and APA transcripts were identified via bedtools intersect with reciprocal overlap. TTS‐associated m6A peaks (±60 nt from APA sites) were further analyzed using deeptools computeMatrix to generate base‐resolution m6A density profiles.

### 2.17 eCLIP‐seq

The enhanced crosslinking and immunoprecipitation (eCLIP) workflow was adapted from established methodologies with a previous protocol.^24^, ^27^ Briefly, OVCAR3 cells expressing CPSF6‐FLAG fusion proteins were subjected to 254 nm UV irradiation and lysed in eCLIP buffer supplemented with RNase inhibitor (Invitrogen EO0382) and Turbo DNase (Invitrogen AM2238). FLAG‐tagged ribonucleoprotein complexes were immunoprecipitated using anti‐FLAG (MBL M185‐3L) and Protein G beads through incubation at 4°C. Captured RNA‐protein complexes underwent sequential enzymatic processing: 3′‐end dephosphorylation with T4 polynucleotide kinase (NEB M0201L), followed by 3′ RNA adapter ligation. Following electrophoresis on 4–12% Bis‐Tris gradient gels, protein‐RNA complexes were transferred to nitrocellulose membranes. Target bands corresponding to protein‐RNA complexes and input controls were excised and digested by proteinase K (Roche). The harvested RNA was reverse‐transcribed using SuperScript III (Thermo Fisher, USA), and cDNA libraries were then purified using a DNA Clean and Concentrator Kit (Zymo Research). Finally, paired‐end sequencing (150 bp) of libraries was performed on an Illumina HiSeq 1000 platform (Novogene). The data analysis processing included read alignment via STAR against GRCh38.p5, peak calling using HOMER (FDR < 0.05, |log2FC| > 6), and functional enrichment analysis using DAVID with GO/KEGG annotations.

### 2.18 Rapid amplification of cDNA 3′ ends (3′ RACE) assay

The SMARTer RACE 5′/3′ Kit (TaKaRa, 634859) was employed to perform the 3′ RACE assay with total RNA. The gene‐specific primer sequences for PUM2, ROCK2, and CELF1 were provided in Table S1. Briefly, RNAs were converted into RACE‐Ready first‐strand cDNA and diluted with Tricine‐EDTA buffer. The primers were used as PCR templates with 3′‐RACE‐ready cDNA for generating the full‐length cDNAs. The 3′RACE products were then analyzed by 1.0% agarose gel electrophoresis.

### 2.19 mRNA stability assay

The control group and knockdown group cells were seeded into 12‐well plates. When the confluence reached 80%, 5 µg/mL actinomycin D (Adooq Bioscience) was added to the cells for the mRNA stability assay. Cells were harvested at different times (0, 2, 4, 6 and 8 h, respectively). Total RNA was extracted using TRIzol reagent and analyzed by RT‐qPCR. The mRNA expression was normalized to that of 18S ribosomal RNA, and linear regression analysis was performed to determine the half‐life of the mRNA.

### 2.20 Dual luciferase reporter assay

The 3′UTR of PUM2 containing the m6A and PAS sites was cloned and inserted into multiple cloning sites in the pMIR‐REPORT vector. The reporter plasmids containing three wild‐type or mutant m6A sites (PUM2 3′UTR‐m6A‐WT and PUM2 3′UTR‐m6A‐MUT, respectively) were cotransfected with the IGF2BP2 or pcDNA3.1 empty vector into OC cells. After 2 days of transfection, the cells were collected, and the activities of Rluc and Fluc were measured using the Dual Luciferase Reporter Assay Kit (TransGen) following the manufacturer's guidelines. The Rluc/Fluc ratio was computed and adjusted to the ratio of the mock‐transfected control.

### 2.21 Gene expression correlation and survival analysis

Gene expression correlations were analyzed using the Cancer Genome Atlas (TCGA) data set of OC tissues. The Spearman method was used to determine the correlation coefficient. The Kaplan–Meier plotter was used to assess the significance of gene expression for clinical outcomes, including overall survival (OS) and progression‐free survival (PFS).

### 2.22 Cell proliferation assay

Posttransfection, screened cells were trypsinized, counted, and seeded into 96‐well plates at 2 × 10^3^ cells/well (100 µL cell suspension per well) with quadruplicate technical replicates per group. The plates were incubated under standard culture conditions for designated time intervals (0, 24, 48, 72, and 96 h). At each timepoint, 10 µL of CCK‐8 reagent (APExBIO) was added to each well, followed by 2 h incubation at 37°C. The optical density (OD) of the cells was then measured at 450 nm using a microplate spectrophotometer (Bio‐Rad). A colony formation test was conducted using 2 × 10^3^ cells in six‐well plates. After a 10‐day growth period, the cells were fixed using 4% paraformaldehyde and stained with 0.5% crystal violet. The colonies were then washed with PBS and counted using ImageJ software.

### 2.23 Cell migration and invasion assays

Posttransfection cells were trypsinized, resuspended in serum‐free medium, and counted. For migration assays, 1 × 10^5^ cells in 200 µL serum‐free medium were seeded into the upper chamber of uncoated Transwell inserts (8 µm pore), while invasion assays utilized Matrigel‐precoated (1:5, BD Biosciences) chambers (polymerized for 1 h at 37°C). The lower chambers received 600 µL complete medium (10% FBS). After 36 h incubation, nonmigrated cells were removed from the upper chamber with cotton swabs. Migrated/invaded cells were fixed with 4% paraformaldehyde (30 min), PBS‐washed, stained with 0.5% crystal violet (1 h), rinsed, air‐dried, and imaged (Nikon). Cell counts were analyzed across five random fields per chamber using Photoshop.

### 2.24 In vivo tumorigenicity and metastasis models

Transfected and screened cells from each group were digested, washed twice with sterile PBS, and resuspended in 600 µL PBS mixed with 300 µL Matrigel matrix (Corning, 356237) under sterile conditions. The cell suspension was maintained on ice and immediately transferred to the animal facility. Subcutaneous injections were performed in the inguinal region of nude mice (BALB/c‐nu, female, 4‐week‐old) using 5 × 10^6^ cells in 100 µL suspension per mouse. Each experimental group contained five mice to ensure statistical power. The xenograft tumour formation was monitored by callipers every week, and the volume was calculated by the formula (smallest diameter^2^ × largest diameter)/2. When the tumour volume reached 1000 mm^3^, the mice were sacrificed, and the tumours were collected, weighed, and analyzed. The xenograft tumours were subjected to IHC and HE staining. On the other hand, to establish the peritoneal metastasis model of OC, Nude mice (BALB/c‐nu, female, 6 weeks) were intraperitoneally injected with 300 µL cell suspension (1 × 10⁶ transfected cells), with five mice per experimental group. Body weights were recorded preinjection and monitored weekly (starting Day 7), followed by triweekly measurements from Days 10–28. After 28 days, mice were euthanized via cervical dislocation under anaesthesia. Thoracoabdominal skin was dissected using ophthalmic instruments to document intraperitoneal tumour growth. All tumour nodules were excised, counted and weighed. Gross morphology and metastatic foci were systematically analyzed. All murine studies were performed at the specific‐pathogen‐free (SPF) Laboratory Animal Center of Chongqing Medical University (CQMU) under strictly controlled environmental conditions. The experimental protocols were reviewed and approved by the CQMU Institutional Animal Care and Use Committee (approval no. IACUC‐CQMU‐2023‐0153).

### 2.25 Statistical analysis

All experiments were repeated at least three times independently. The data are presented as the mean ± standard deviation (SD), and GraphPad Prism software (GraphPad 6.0) was used for partial statistical analyses. A Student's *t*‐test determined the statistical significance between two groups, and a one‐way ANOVA was conducted for comparisons among three or more groups. The Kaplan–Meier method and the log‐rank test were applied to estimate PFS, OS, and their differences. The *p*‐value < 0.05 was considered statistically significant.

## 3 RESULTS

### 3.1 The relationship between m6A and APA in OC

**FIGURE 1:**
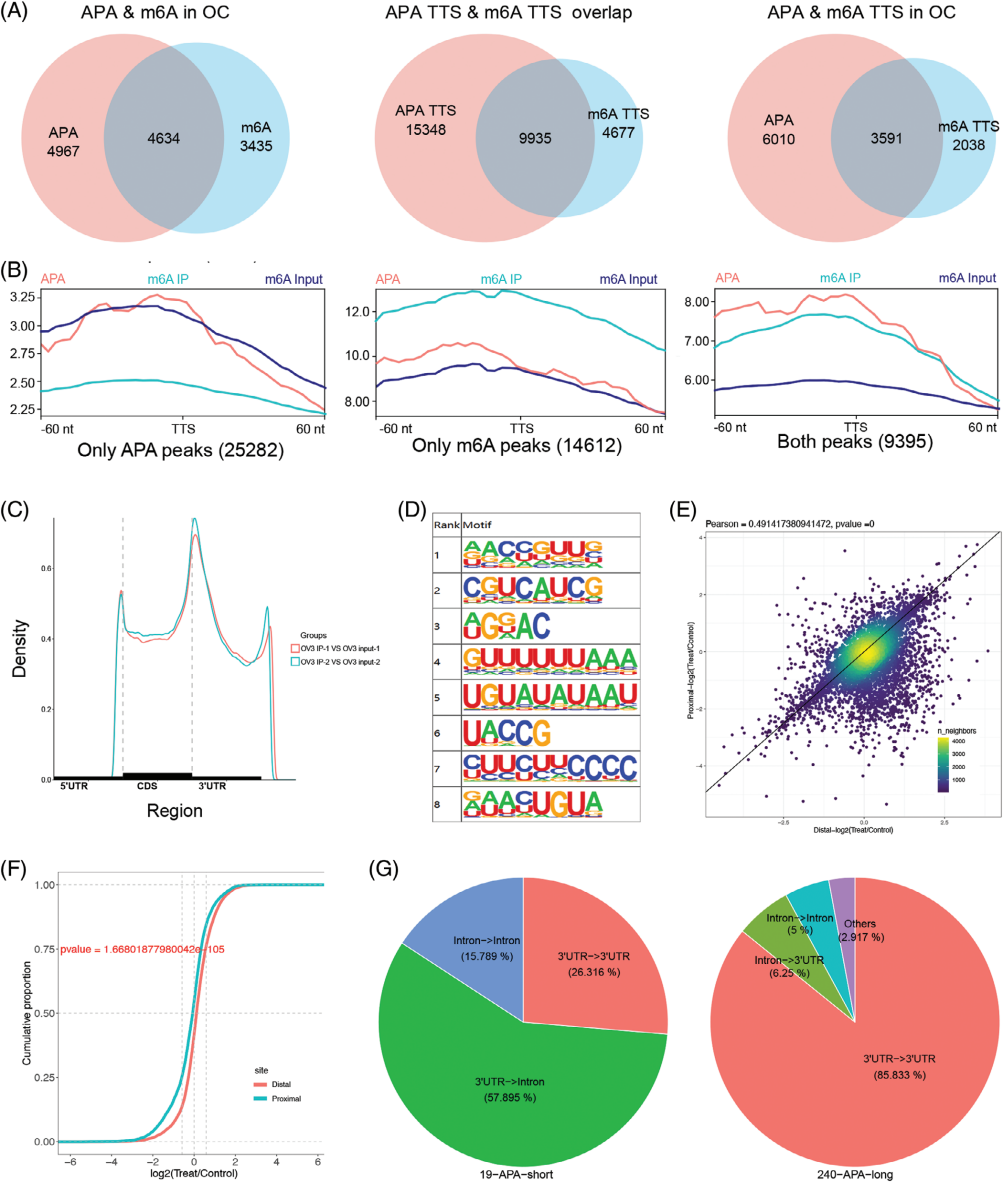
The relationship between m6A and APA in OC. (A) Combined analysis of meRIP‐seq and scRNA‐seq of APA data in OC. (B) Peak analysis of m6A modification and APA processing. (C) The peak map of meRIP‐seq. (D) The binding motif of meRIP‐seq. (E, F) PAS‐seq analysis of METTL3 knockdown in OVCAR3, the volcano map of long and short genes (E), and the proximal and distal PAS uses S‐shaped curve (F). (G) The PAS distribution of APA genes after METTL3 knockdown.

Based on our previous research about APA processing from scRNA‐seq of OC tissues (GSE154600),^24^, ^28^ we performed meRIP‐seq on OVCAR3 cells and obtained m6A modification data. Taking the intersection of genes with m6A modification in the meRIP‐seq data and genes with APA dynamics in the scRNA‐seq data, we found that 48.27% (4634/4634 + 4967) of APA transcripts had m6A modification. Analyzing its position on the transcript, it was found that there were 9935 peaks falling in the TTS region of 3591 genes (Figure 1A). During further analysis of the sequencing data, we also found that the m6A sites were very close to or even overlapped with the APA TTS region (Figure 1B). These results suggest that m6A‐modification transcripts could have more frequent APA phenomenon. For meRIP‐seq, the meRIP‐seq showed that m6A peaks were enriched at start codons, stop codons and 3′UTRs (Figure 1C), and the m6A consensus sequence “GGAC” was the most common motif (Figure 1D). Moreover, we observed that the APA‐specific motif “UGUA” was enriched according to the meRIP‐Seq (Figure 1D). These results suggested that m6A may be associated with APA processing.

To further assess the effect of m6A on APA regulation, we knocked down the expression of METTL3, which is the predominant catalytic subunit of m6A methyltransferase, and PAS‐seq was performed in OVCAR3 cells (Figure S3A,B). A total of 259 differential APA genes were identified; 240 genes were lengthened and 19 genes were shortened in OVCAR3 cells (Figure 1E; Table S4). Knockdown of METTL3 resulted in a significant increase in the distal PAS usage of APA genes (Figure 1F). The PASs of the APA genes primarily changed from 3′UTRs to 3′UTRs and 3′UTRs to introns (Figure 1G). Therefore, we found that the m6A methyltransferase METTL3 could affect the selection and usage of PAS in OC.

### 3.2 Identification of the interactions between APA factors and m6A regulators

**FIGURE 2:**
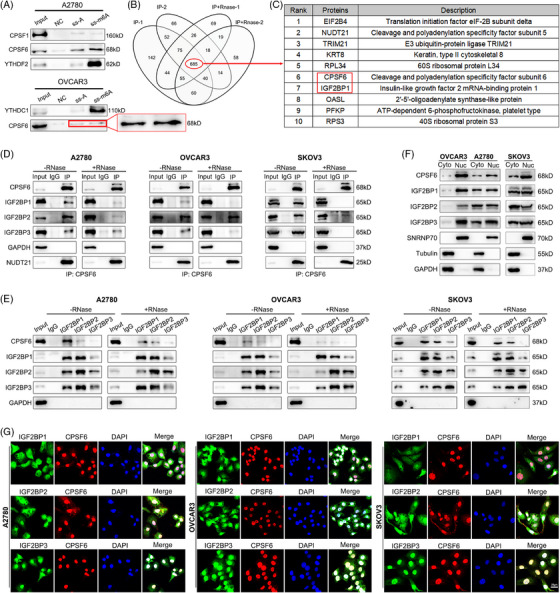
The APA factor CPSF6 interacts with the m6A regulator IGF2BP1/2/3 complex in OC cells. (A) CPSF6 was related to m6A modification according to the RNA‒protein pulldown assay. (B) The binding proteins of CPSF6 were detected by IP/MS in OVCAR3 cells. (C) List of the top 10 proteins for CPSF6‐IP. (D) IP assay using a CPSF6 antibody with or without RNaseA to detect the IGF2BP1, IGF2BP2 and IGF2BP3 proteins in OC cells. (E) IP assay of IGF2BP1/2/3 with or without RNaseA to detect CPSF6. (F) The distribution of proteins was detected by nucleocytoplasmic separation. (G) The colocalization of the IGF2BP1/2/3 complex and CPSF6 was detected by an immunofluorescence assay.

We speculated that m6A modification might regulate the APA process by interacting with APA core factors in OC. To detect which APA factor is related to m6A, we used methylated single‐stranded RNA bait (ss‐m6A) or unmethylated control RNA (ss‐A) for the RNA‒protein pulldown assay. Through an experimental screen for the core regulatory factors of APA, we identified that only CPSF6 selectively bound to the methylated bait (ss‐m6A) with greater affinity than did the unmethylated control (ss‐A) in OC cells (A2780 and OVCAR3) (Figure 2A; Figure S1A), and the affinities of the other regulators did not significantly differ (Figure S1A). In a previous study, Yue et al.^29^ reported that VIRMA is associated with NUDT21 and CPSF6 in an RNA‐dependent manner. We repeated the protein IP assay in HeLa cells and found that the m6A writers VIRMA, MELLT3, and METTL14 were associated with NUDT21 and CPSF6 (Figure S1B). Subsequently, we tested protein interactions in OVCAR3 cells and found that VIRMA, MELLT3, and METTL14 interacted with NUDT21 and CPSF6 in an RNA‐dependent manner, indicating that some mRNAs contain both m6A modification and APA processing and that m6A methyltransferases may indirectly participate in the regulation of APA combination (Figure S1C). In turn, we performed IP to examine the interactions between CPSF6 and m6A‐related proteins within the nucleus, which mainly included methyltransferases (VIRMA, WTAP, MELLT3, and METTL14), demethyltransferases (FTO and ALKBH5) and recognition proteins (YTHDC1, HnRNPC, and HnRNPA2B1). The results showed that CPSF6 had no obvious binding with these proteins in HeLa, A2780 or OVCAR3 cells (Figure S1D,E). To further explore the relationship between CPSF6 and m6A, we performed CPSF6 IP‐MS with or without RNase A treatment in OVCAR3 cells (Figure S1F). Using the intersection of the results, 685 common proteins were selected for further analysis (Figure 2B; Table S2). Consistent with previous studies, many reported CPSF6 binding proteins, such as NUDT21, CPSF7, SRSF3, SRSF7, SRSF10, TRA2B, and NXF1, were identified, suggesting that the MS data are reliable and informative.^30^, ^31^ These candidate proteins were enriched in ribosome, RNA transport, spliceosome and RNA catabolic process (Figure S1G). The list contained multiple m6A‐related proteins (METTL3, IGF2BP1, IGF2BP2, IGF2BP3, YTHDF2, and HNRNPC), and IGF2BP1 was among the top 10 (Figure 2C). Based on the results of the above sections, we performed a CPSF6 IP assay with or without RNase A treatment in OC cells (A2780, OVCAR3, and SKOV3), and the results showed that CPSF6 could directly bind to the IGF2BP1, IGF2BP2, and IGF2BP3 proteins (Figure 2D). On the other hand, IGF2BP1, IGF2BP2, and IGF2BP3 IP assays revealed that the IGF2BP1/2/3 complex also interacted with the CPSF6 protein in an RNA‐independent manner in OC cells (Figure 2E). According to previous reports, CPSF6 mainly regulates APA processing and alternative splicing of pre‐mRNAs in the nucleus, and IGF2BP1/2/3 impacts mRNA stability and translation in the cytoplasm.^25^, ^31^ To characterize the location of the association between CPSF6 and IGF2BP1/2/3 proteins in OC cells, we performed nuclear and cytoplasmic protein extraction and immunofluorescence assays. The CPSF6 and IGF2BP1/2/3 proteins were distributed in both the cytoplasm and nucleus in A2780 and SKOV3 cells, and they mainly resided in the nucleus in OVCAR3 cells (Figure 2F). There was significant colocalization of the CPSF6 and IGF2BP2/3 proteins in OC cells, as shown by immunofluorescence staining (Figure 2G). These results confirmed the interaction between the APA factor CPSF6 and the m6A regulator complex IGF2BP1/2/3 in OC.

### 3.3 The CPSF6 RS domain directly interacts with the C‐terminal domain of IGF2BP2

**FIGURE 3:**
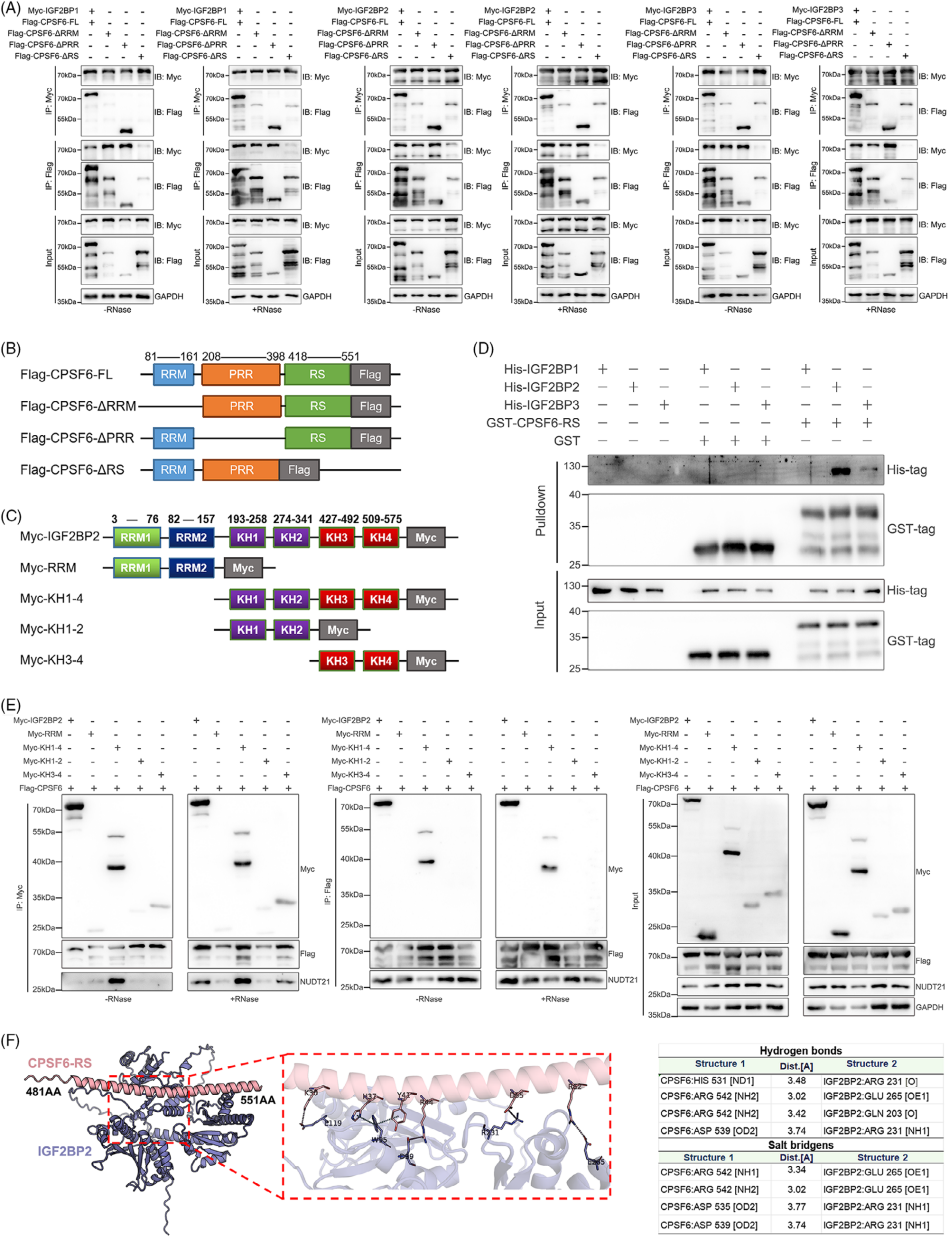
CPSF6 RS domain directly binds to the C‐terminal KH domains of IGF2BP2. (A) Co‐IP assay of Flag‐CPSF6 truncated and Myc‐IGF2BP1/2/3 in 293T cells. (B, C) Schematic diagram of CPSF6 truncated (B) and IGF2BP2 truncated (C) proteins. (D) Western blot analysis of GST‐CPSF6‐RS or His‐IGF2BP1/2/3 proteins of GST‐input and pulldown samples. (E) Co‐IP assay of Myc‐IGF2BP2 truncated and Flag‐CPSF6 in 293T cells. (F) The structure and site of the CPSF6 RS‐IGF2BP2 interaction complex were predicted by AlphaFold3.

To further investigate the domains for IGF2BP1/2/3 binding in CPSF6, we constructed FLAG‐tag plasmids with the full‐length (FL) recombinant human CPSF6 protein and several truncated proteins (ΔRRM, ΔPRR, and ΔRS) from the UniProt database (Figure 3B). First, we examined the relationship between truncated CPSF6 proteins and the IGF2BP1/2/3 complex using an IP assay with or without RNase A treatment in OC cells. When RNase A was not added, the truncated ΔRRM, ΔPRR, and ΔRS proteins displayed similar binding with IGF2BP1/2/3 as the full‐length protein of CPSF6 in OC cells. Strikingly, the intensity of the ΔRS signal was abolished in the OVCAR3 cells treated with RNase A (Figure S2A,B). There was a significant decrease in NUDT21 expression in response to IP of the CPSF6 ΔRRM protein, which was consistent with previous observations.^31^ Therefore, we predict that the RS domain of CPSF6 may play a dominant role in binding to IGF2BP1/2/3 in OC cells. To further clarify the binding domain, we constructed Myc‐IGF2BP1, Myc‐IGF2BP2 and Myc‐IGF2BP3 plasmids and performed coimmunoprecipitation (Co‐IP) experiments with Myc‐tag and FLAG‐tag as baits without or within RNase A by co‐transfecting the Myc‐IGF2BP1/2/3 and CPSF6 (FL and truncation) plasmids, respectively, into 293T cells. The results showed that the proteins Myc‐IGF2BP1, Myc‐IGF2BP2, and Myc‐IGF2BP3 could pull down FLAG‐CPSF6 in the presence and absence of RNA; notably, the densities of the ΔRRM and ΔRS strains were lower than those of the FL and ΔPRR strains, which were combined with IGF2BP1/2/3 (Figure 3A). Meanwhile, the FLAG‐CPSF6 (FL, ΔRRM, ΔPRR, and ΔRS) proteins also precipitated Myc‐IGF2BP1/2/3; however, the ability of ΔRS to bind IGF2BPs was significantly lower than that of FL, ΔRRM, and ΔPRR (Figure 3A). We deduced that the CPSF6 RS domain was the core region involved in direct binding to the IGF2BP1/2/3 complex.

The IGF2BP1, IGF2BP2, and IGF2BP3 proteins constitute a protein complex, and we wondered which IGF2BP proteins (IGF2BP1, IGF2BP2, and IGF2BP3) directly bind to the CPSF6 protein. We purified the human His‐IGF2BP1, His‐IGF2BP2, His‐IGF2BP3, and GST‐CPSF6‐RS proteins and applied a GST pulldown assay in vitro to determine their interactions (Figure S2C). The recombinant IGF2BP2 protein was significantly pulled down by the GST‐CPSF6‐RS fusion protein (Figure 3D), which indicated that the RS domain of CPSF6 directly bound to the IGF2BP2 protein. IGF2BP2 contains dual N‐terminal RNA recognition motifs (RRM1/RRM2, residues 3–157) mediating mRNA binding through conserved RNP1/RNP2 sequences, and four C‐terminal K homology domains (KH1‐KH4, residues 193–575) that stereospecifically recognize m6A‐modified RNAs via conserved GXXG motif interactions.^25^ To spatially resolve CPSF6‐binding epitopes, we engineered Myc‐tagged truncations: RRM, KH1‐4, KH1‐2, and KH3‐4 (Figure 3C). Co‐IP assays of 293T cells cotransfected with the CPSF6 and IGF2BP2 truncation plasmids with or without RNase A treatment suggested that CPSF6 could bind to the C‐terminal domain of IGF2BP2 but not the RRM domain (Figure 3E). Furthermore, protein–protein interaction predicts the presence of 4 hydrogen bond connections and four salt bridge structures between CPSF6 RS and IGF2BP2 KH domain (Figure 3F). The above results verified direct binding between the CPSF6 RS domain and the IGF2BP2 KH domain.

### 3.4 IGF2BP2 regulates APA by recruiting CPSF6 in an m6A‐dependent manner

**FIGURE 4:**
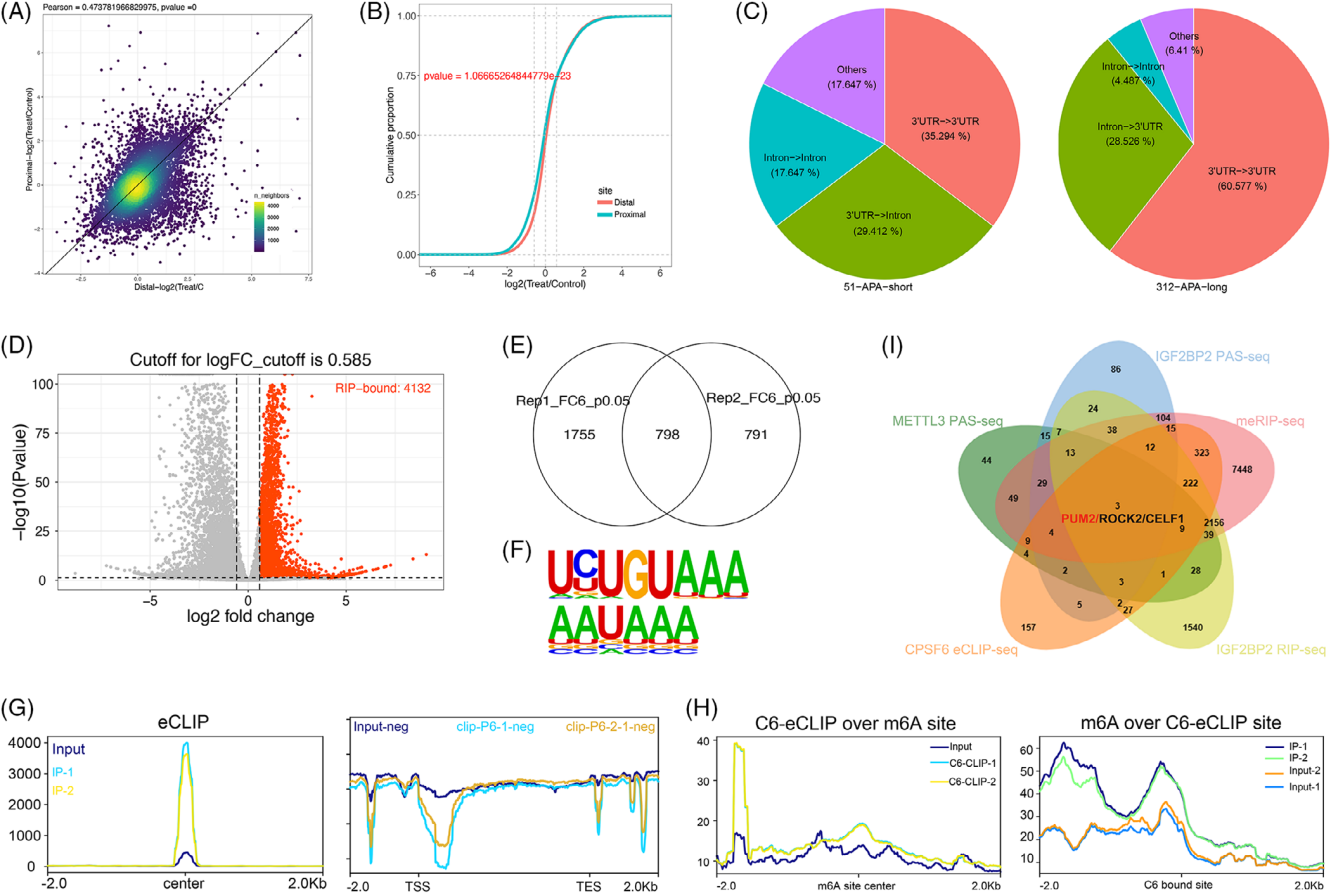
Joint analysis of multiple sequencing for screening target genes in OVCAR3 cells. (A, B) PAS‐seq analysis of IGF2BP2 knockdown in OVCAR3, the volcano map of long and short genes (A), and the proximal and distal PAS uses a S‐shaped curve (B). (C) The PAS distribution of short and long APA genes after IGF2BP2 knockdown. (D) The volcano plot of IGF2BP2 binding genes by RIP‐seq. (E) Overlap of the CPSF6 binding target genes. (F) The enrichment motif analysis of CPSF6 eCLIP‐seq. (G) Venn diagram showing the overlap of the multiple sequencing results. (H) The peak map of CPSF6 binding sites. (I) Joint analysis of m6A‐seq and CPSF6 eCLIP‐seq.

We hypothesize that IGF2BP2 may mediate the regulation of m6A and APA by interacting with CPSF6 in OC cells. To investigate the role of IGF2BP2 in global APA, we knocked down the expression of IGF2BP2 and performed PAS‐seq in OVCAR3 cells (Figure 3A,B). Through PAS‐seq analysis, we identified genes with significantly different 3′UTR lengths between the control and IGF2BP2 knockdown samples; 312 genes with extended 3′UTRs and 51 genes with shortened 3′UTRs were identified (Figure 4A; Table S3). Moreover, IGF2BP2 knockdown favoured the use of the distal PAS in target genes (Figure 4B). The alternative sites of the APA genes were primarily located in the 3′UTR to 3′UTR and intron to 3′UTR regions (Figure 4C). In addition, these APA genes following METTL3 and IGF2BP2 knockdown were enriched in several cancer‐related pathways, such as the Hippo pathway, autophagy, the TGF‐beta pathway, the AMPK pathway and cancers (Figure S3C,D). Taken together, the PAS‐seq results showed that knocking down m6A regulators (METTL3 and IGF2BP2) increased the use of the distal PAS to lengthen the APA genes in OC cells.

To identify IGF2BP2‐bound RNAs, we employed IGF2BP2 RIP‐seq in OVCAR3 cells. The data analyses revealed that 4132 genes were bound by IGF2BP2 (Figure 4D; Table S6) and were enriched in several cancer pathways, including the MAPK pathway, Wnt pathway, Hippo pathway, and Notch pathway (Figure S3F). Then, we performed eCLIP‐seq analysis in OVCAR3 cells and mapped the binding sites of CPSF6 to its target RNAs (Figure S3G). 798 target genes of CPSF6 were screened (Figure 4E; Table S7), most of which were protein‐coding genes, and the binding sites were located mainly in the intronic and 3′UTR regions of the target genes (Figure S3H). The analysis showed that the canonical PAS motifs “AAUAAA” and “UGUA” were enriched in the targets of CPSF6 (Figure 4F), which was consistent with a previous report showing that the AAUAAA sequence is recognized by the CPSF complex and that the UGUA motif is bound to the CFIm complex.^23^ The peaks were predominantly localized near transcription start sites (TSSs) and transcription end sites (TESs) (Figure 4G). Furthermore, we integrated m6A‐seq with eCLIP‐seq analysis and revealed that the degree of CPSF6 binding was greater near the m6A sites and that the m6A modification was closer to the binding site of CPSF6 than in the input groups (Figure 4H), indicating that there was a specific interaction between m6A and APA mediated by CPSF6. To better understand the functions of the target genes, we performed GO biological function and KEGG pathway enrichment analyses. The GO terms were enriched in mRNA metabolic process, histone modification, nuclear speck, cytoplasmic stress granule and transcription coregulator activity, and the KEGG pathways implicated several cancer pathways, such as the mTOR pathway, Wnt pathway, autophagy, AMPK pathway, TGF‐beta pathway, and Notch pathway (Figure S3I). Finally, after overlapping the genes from the PAS‐seq, meRIP‐seq, RIP‐seq and eCLIP‐seq data, three genes (PUM2, ROCK2, and CELF1) bound to IGF2BP2 and CPSF6 and were coregulated by m6A and APA (Figure 4I; Table S8). PAS‐seq analysis revealed that the lengths of PUM2, ROCK2, and CELF1 were increased when METTL3 and IGF2BP2 were knocked down (Figure S3J). Consequently, we speculate that the m6A modifications of these genes catalyzed by METTL3 are recognized by IGF2BP2, after which IGF2BP2 directly binds to CPSF6, facilitating the use of the proximal PAS and resulting in shorter lengths of target genes and affecting RNA stability, translation efficiency or protein expression.

### 3.5 METTL3 and IGF2BP2 regulate the APA process to affect the stability of PUM2 with m6A‐methylated and CPSF6‐bound

**FIGURE 5:**
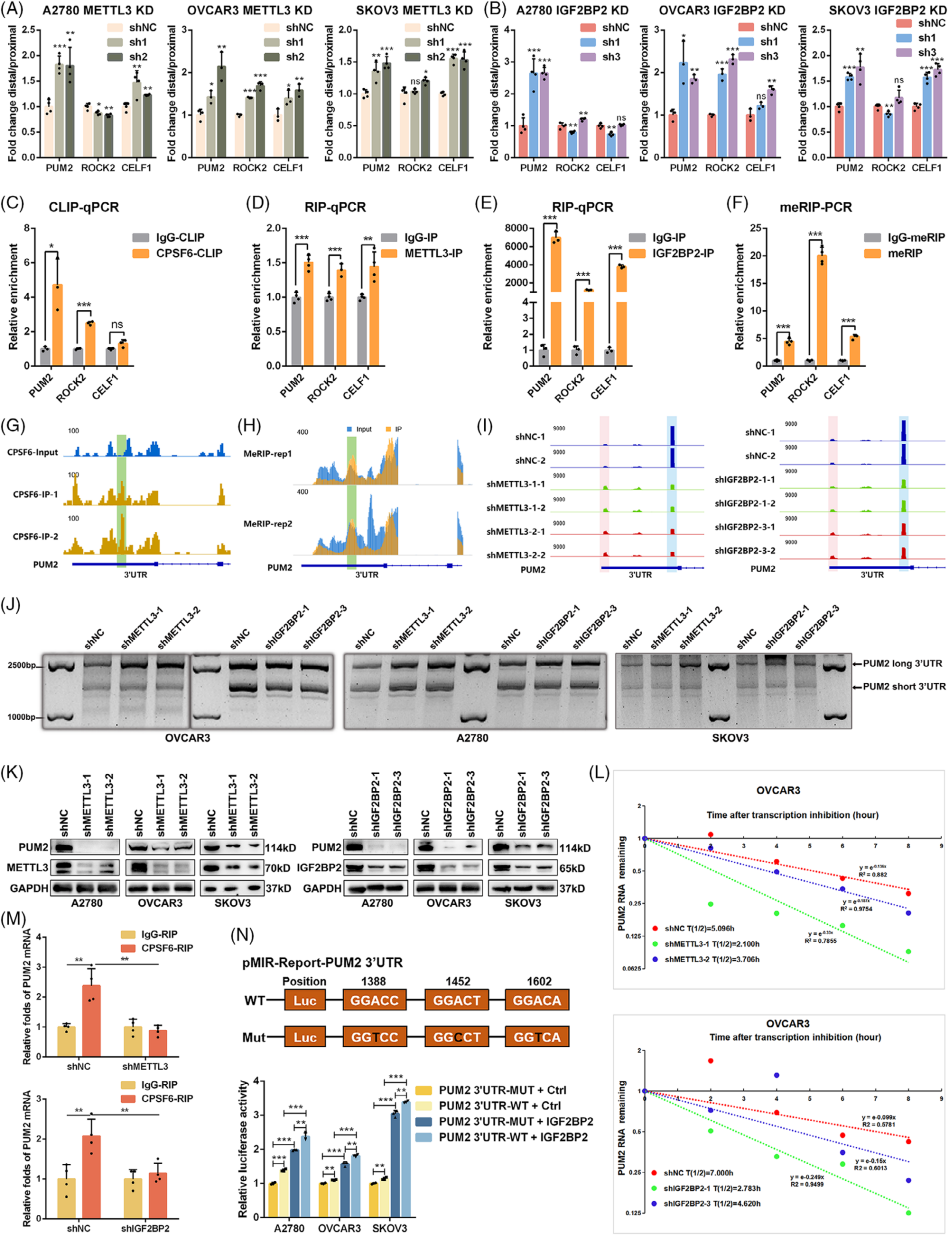
METTL3 and IGF2BP2 regulate the APA process to affect the stability of PUM2 with m6A‐dependent and CPSF6‐binding manner in OC cells. (A, B) The expression ratio of long and short transcripts of target genes after knockdown of METTL3 (A) and IGF2BP2 (B) in OC cells, respectively. (C–F) The interaction between 3′UTR of PUM2 mRNA and CPSF6 (C), METTL3 (D), IGF2BP2 (E), and m6A (F) antibodies was validated by using eCLIP‐seq, RIP‐PCR and meRIP‐seq. (G) Distribution of the CPSF6 binding sites across PUM2 mRNA transcript as identified by eCLIP‐seq. (H) Distribution of m6A peaks across PUM2 mRNA as identified by meRIP‐seq. (I) IGV tracks showing the enrichment of PUM2 mRNA according to PAS‐seq after METTL3 and IGF2BP2 knockdown in OVCAR3 cells. (J) The abundance of PUM2 long and short transcripts was identified by 3′RACE assay in OC cells with METTL3 or IGF2BP2 knockdown. (K) The expression of PUM2 was detected by western blot after knocking down METTL3 and IGF2BP2 in OC cells. (L) Decay curves and half‐life (T1/2) of PUM2 mRNA in OVCAR3 cells with METTL3 and IGF2BP2 knockdown were derived from mRNA stability profiling. (M) The interaction between CPSF6 and PUM2 mRNA was detected by RIP‐qPCR following METTL3 and IGF2BP2 knockdown in OVCAR3 cells. (N) Scheme of m6A sites WT and MUT nucleotide sequence surrounding PUM2 pPAS in 3′UTR, and dual luciferase reporter assay performed by cotransfecting PUM2 3′UTR WT or MUT and IGF2BP2 plasmid.

To test the changes in the length of target genes, we designed two pairs of primers to quantify the expression of the common and extended regions of the 3′UTR. The ratio of extended/common represented the PAS usage between distal and proximal mRNA levels. Compared with those of the control, the ratios (distal/proximal) of three target genes (PUM2, ROCK2 and CELF1) were increased when METTL3 and IGF2BP2 were downregulated in OC cells (Figure 5A,B), especially for the PUM2 mRNA. This result indicated that the longer transcripts of target genes were upregulated, which was consistent with the PAS‐seq results. Next, to validate the results of eCLIP‐seq, RIP‐seq and meRIP‐seq, the target genes were chosen for CLIP‐qPCR, RIP‐qPCR, and meRIP‐qPCR analysis, respectively (Figure S4A). Based on the CPSF6 binding sites from eCLIP‐seq, we designed primers for the CLIP‐qPCR assays and found that the relative enrichment of PUM2 and ROCK2 was significantly greater than that in the negative control, but CELF1 was not significantly enriched in the OVCAR3 cells (Figure 5C). Following RIP‐qPCR analysis of METTL3 and IGF2BP2, three target genes, particularly PUM2 in the IGF2BP2‐IP system, were found to be enriched relative to those in the IgG antibody‐treated group (Figure 5D,E). The results of meRIP‐qPCR confirmed that the relative m6A modifications of the three target genes were significantly greater than those of the negative control (Figure 5F). Moreover, the peaks of the CPSF6 eCLIP and m6A signals at the proximal PAS of the PUM3 3′UTR fully overlapped and were confirmed by RT‐qPCR assays in OVCAR3 cells (Figure 5G,H). Meanwhile, employing the Integrative Genomics Viewer (IGV) browser to examine PAS‐seq data, we observed a reduction in the use of the PUM2 proximal PAS and a notable increase in the distal PAS usage when METTL3 and IGF2BP2 were silenced in OVCAR3 cells (Figure 5I). On the other hand, to verify the switch from proximal to distal PAS usage and the extension of the 3′UTRs of target genes caused by METTL3 and IGF2BP2 knockdown, we performed a 3′RACE assay to detect the transcriptional variants of the PUM2, ROCK2, and CELF1 3′UTRs in OC cells. The results revealed two distinct 3′UTR isoforms of PUM2: the short isoform was decreased, and the long isoform was increased in OC cells following METTL3 and IGF2BP2 knockdown (Figure 5J). However, the transcript levels of ROCK2 and CELF1 did not significantly change in OC cells with METTL3 and IGF2BP2 knockdown compared with those in the controls, and ROCK2 had multiple transcripts with different 3′UTRs (Figure S4B). According to the alignment positions and experimental results, PUM2 was selected as the representative target gene, and its 3′UTR was the pivotal region for downstream investigation.

To further test the impact of m6A on PUM2, we examined the effects of METTL3 and IGF2BP2 on the expression of PUM2. The western blot results showed that downregulation of METTL3 and IGF2BP2 reduced the expression level of the PUM2 protein in OC cells (Figure 5K). As we know from earlier studies, mRNA 3′UTRs contain many miRNA regulatory sites, cis‐RNA regulatory elements, trans‐acting RNA‐binding protein (RBP) sites, and other regulatory sequences, which influence mRNA abundance and translation.^32^ A shortened 3′UTR with loss of negative regulation at these sites generally increases mRNA stability and translation efficiency.^33^ To further reveal the relationship between the expression and 3′UTR length of PUM2 in our research, we examined the mRNA stability of PUM2 by silencing METTL3 and IGF2BP2 in OC cells. Our results showed that METTL3 and IGF2BP2 knockdown decreased the stability of the PUM2 gene (Figure 5L; Figure S4C,D). In addition to genetic knockdown, we treated cells with METTL3 inhibitor STM2457 and IGF2BP2 inhibitor CWI1‐2. Both inhibitors significantly reduced PUM2 protein levels (Figure S4E). Thus, the above results showed that the longer isoform of PUM2 had lower mRNA stability and protein expression after interfering with the expression of METTL3 and IGF2BP2 in OC cells.

Because knocking down METTL3 or IGF2BP2 promoted the use of the PUM2 distal PAS and because IGF2BP2 directly interacted with CPSF6, we next tested whether METTL3/IGF2BP2‐mediated m6A modification affects the APA of the PUM2 gene via CPSF6. We performed RIP‐qPCR assays using CPSF6 via METTL3 or IGF2BP2 knockdown (Figure S4F) and found that METTL3 or IGF2BP2 knockdown markedly reduced the binding of CPSF6 to PUM2 transcripts in OVCAR3 cells (Figure 5M). To further confirm the regulatory mechanism of m6A and APA, the proximal PAS of the PUM2 3′UTR with embedded m6A modification sites and the mutated m6A sites were cloned and inserted into a luciferase reporter. We cotransfected IGF2BP2 or the pDCNA3.1 vector with PUM2 3′UTR‐WT and PUM2 3′UTR‐Mut. The results showed that the overexpression of IGF2BP2 significantly increased the reporter activity of the PUM2 3′UTR‐WT and PUM2 3′UTR‐Mut compared with that of the vector, and the reporter activity of the PUM2 3′UTR‐WT group was greater than that of the PUM2 3′UTR‐Mut group in OC cells (Figure 5N). To examine broader m6A networks, we found that FTO knockdown upregulated PUM2 expression (Figure S4G). And YTHDF1/2 knockdown did not alter PUM2 expression (Figure S4H,I), confirming the specificity of IGF2BP2 in this regulatory axis. Thus, our results confirmed that IGF2BP2 promotes the proximal PAS usage of PUM2 by accelerating the function of CPSF6 in an m6A‐dependent manner.

### 3.6 IGF2BP2 promotes OC proliferation and metastasis

Both METTL3 and IGF2BP2 play oncogenic roles in OC, and the molecular and cellular roles of METTL3 in OC patients have been well documented in previous studies.^34^ However, there are few reports on the function of IGF2BP2 in OC, and the biological mechanism of IGF2BP2 in OC is largely undetermined. Thus, we further explored the functions and mechanisms of IGF2BP2 in the progression of OC. To clarify the role of IGF2BP2 in OC, we performed loss‐of‐function experiments using shRNAs both in vitro and in vivo. Through CCK‐8 and colony formation assays, we showed that reducing IGF2BP2 inhibited the proliferation of OC cell lines in vitro (Figure S5A,B). Transwell assays demonstrated that knockdown of IGF2BP2 inhibited the migration and invasion of OC cells in vitro (Figure 5C). Moreover, the subcutaneous tumour transplant model further verified that IGF2BP2 knockdown decreased the growth of OVCAR3 xenografts in vivo (Figure S5D–F). In addition, an abdominal cavity metastasis mouse model generated by intraperitoneal injection of OVCAR3 cells showed that IGF2BP2 knockdown attenuated peritoneal cavity metastasis in vivo (Figure S5G). Moreover, the expression of Ki‐67 (a proliferative marker) and Caspase‐3 (an apoptotic marker) in OVCAR3 xenograft tumours was examined by IHC. The results showed that Ki67 was decreased and Caspase‐3 was increased in the IGF2BP2 knockdown group compared with the control group (Figure S5H). These results indicate that IGF2BP2 acts as an oncogene to promote the progression of OC.

### 3.7 PUM2 promotes the progression of OC and is related to METTL3/IGF2BP2 regulation

**FIGURE 6:**
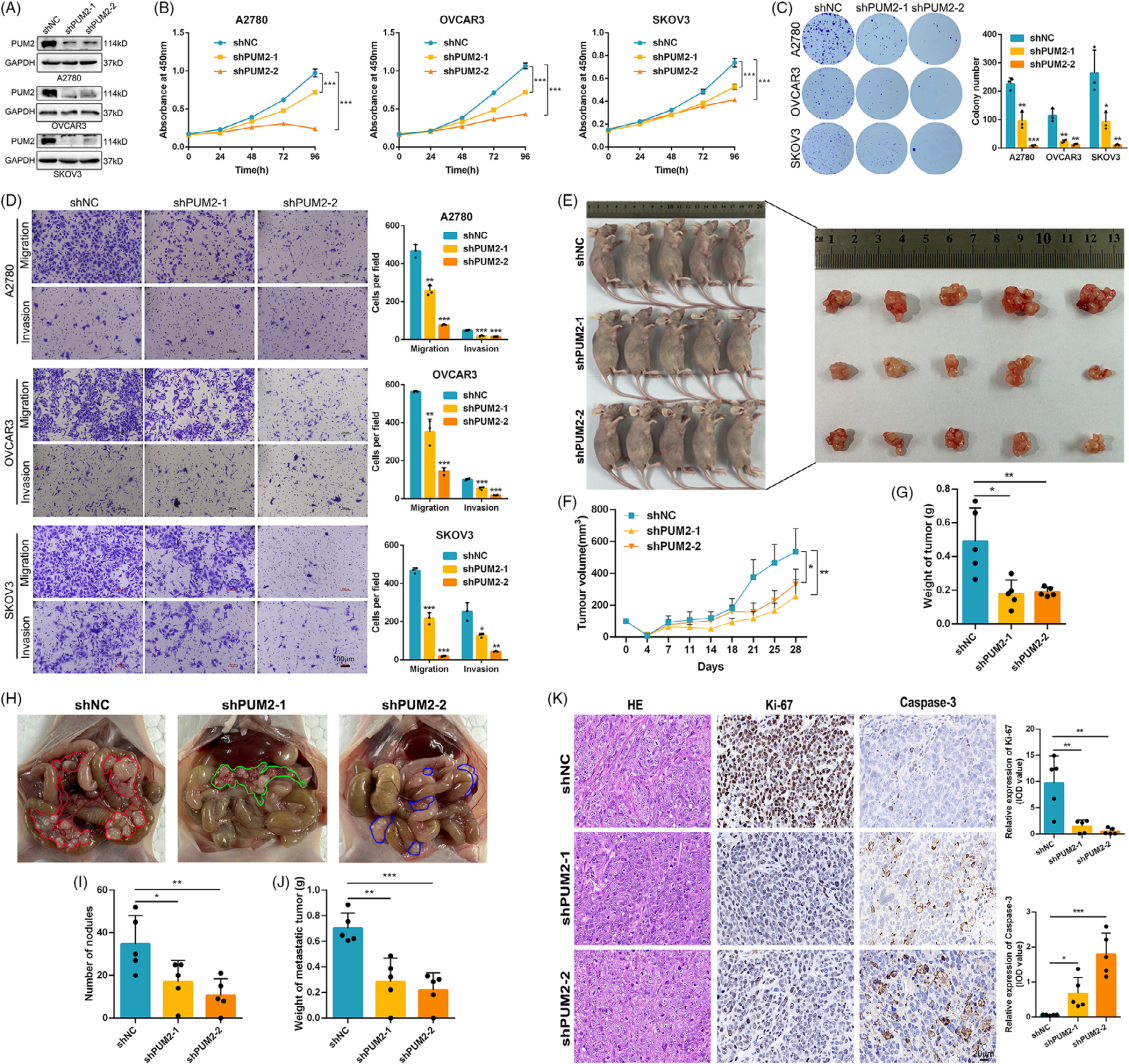
Downregulation of PUM2 expression inhibits the growth and metastasis of OC cells in vitro and in vivo. (A) The knockdown efficiency of PUM2 in OC cells was determined by western blot. (B–D) The growth, proliferation, and metastasis of OC cells were detected by CCK‐8 (B), colony formation (C) and Transwell (D) assays after knocking down PUM2. (E–G) The subcutaneous tumorigenesis model of gross xenograft anatomy (E), growth curve (F) and tumour weight (G) were tested in which PUM2 was knocked down in OVCAR3 cells. (H–J) For the intraperitoneal metastasis model, PUM2‐knockdown OVCAR3 cells were injected into nude mice, and the graphs show the gross anatomy of the abdominal cavity (H), number of metastatic nodules (I) and metastatic tumour weight data (J). (K) HE staining was performed to evaluate tissue morphology, and IHC was performed to visualize Ki‐67‐ and Caspase‐3‐positive staining in xenografted tumours. *p < 0.05, **p < 0.01, ***p < 0.001.

As described above, we hypothesized that IGF2BP2 has carcinogenic effects at least in part by influencing APA processing. The APA target gene PUM2 was directly bound and regulated by IGF2BP2 in our study, but the function of PUM2 in OC has not been reported. We then investigated the functions of PUM2 with the phenotypes of OC cells by downregulating PUM2 expression (Figure 6A). The CCK‐8, colony formation, and Transwell assays indicated that reducing PUM2 expression greatly suppressed the proliferation, migration, and invasion of OC cells. (Figure 6B–D). Furthermore, tumour xenografts in nude mice with OVCAR3 cells revealed that PUM2 knockdown suppressed tumour growth in vivo (Figure 6E–G). Abdominal cavity metastasis in mouse models also indicated that knockdown of PUM2 suppressed the metastasis of OVCAR3 cells in vivo (Figure 6H–J). In addition, an IHC analysis demonstrated that PUM2 knockdown decreased Ki‐67 levels and increased Caspase‐3 expression in OVCAR3 xenografts (Figure 6K). Collectively, our results suggest that, similar to IGF2BP2, PUM2 functions as a crucial oncogene to promote the growth and metastasis of OC in vitro and in vivo.

**FIGURE 7:**
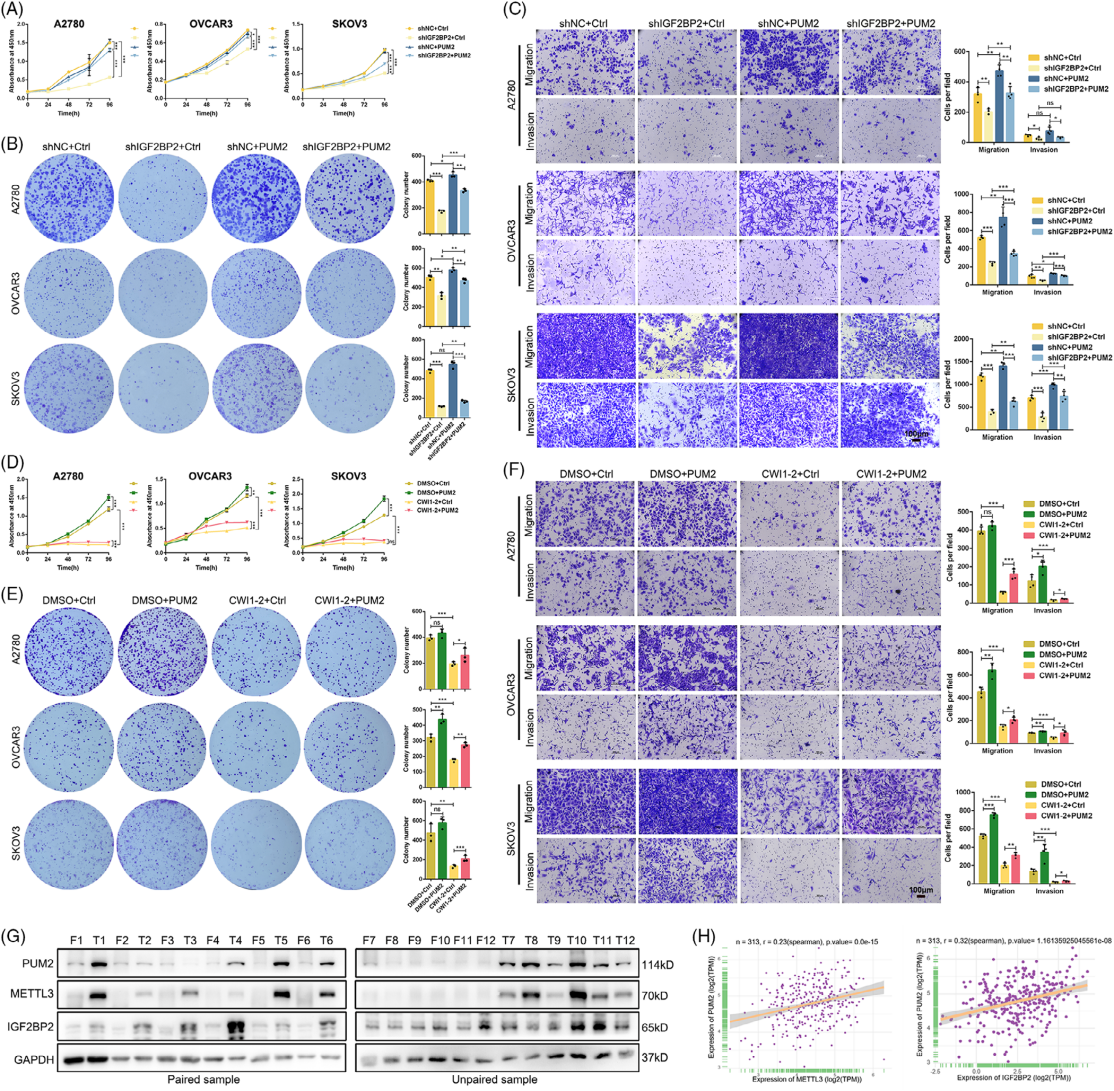
PUM2 serves as a functionally essential target of IGF2BP2 in OC cells. (A–C) The ability of growth, proliferation, and metastasis was detected using CCK8 (A), clone formation (B) and transwell (C) when knockdown IGF2BP2 in OC cells and then overexpressing PUM2. (D–F) Overexpression of PUM2 reversed the growth (D), proliferation (E), and metastasis (F) of OC cells in which IGF2BP2 inhibitor CWI1‐2 treatment. (G) Detection of protein expression levels of PUM2, METTL3, and IGF2BP2 in 12 pairs of OC and normal fallopian tube epithelial tissue samples. (H) Pearson's correlation plot of the expression of METTL3 and IGF2BP2 with that of PUM2 in OC patients from the TCGA database. *p < 0.05, **p < 0.01, ***p < 0.001. ns, not significant.

To evaluate the contribution of PUM2 to the function of IGF2BP2, we conducted a rescue assay by overexpressing PUM2 in IGF2BP2‐knockdown OC cells (Figure S6A). The results showed that restoring the expression of PUM2 substantially reversed the inhibitory effects mediated by IGF2BP2 knockdown in OC cells, including the suppression of cell proliferation, migration and invasion (Figure 7A–C). Subsequently, to functionally validate PUM2 as the critical downstream effector of IGF2BP2 in OC progression, rescue experiments were performed in OC cells treated with the IGF2BP2 inhibitor CWI1‐2 alongside PUM2 overexpression (Figure S6B). CWI1‐2 treatment alone reduced cell viability, suppressed colony formation and decreased migration/invasion of OC cells. Strikingly, PUM2 overexpression significantly reversed these inhibitory effects, restoring proliferation, colony counts and migration/invasion capacity (Figure 7D–F). To further validate the functional hierarchy of the IGF2BP2‐PUM2 axis, reciprocal rescue experiments were performed in OC cells co‐transfected with IGF2BP2 overexpression and lentiviral‐mediated PUM2 knockdown (Figure S6C). Silencing PUM2 attenuated the IGF2BP2 protumorigenic effect, including growth and metastasis (Figure S6D–F). These complementary findings conclusively established that PUM2 is both necessary and sufficient to mediate IGF2BP2‐driven oncogenic phenotypes, solidifying its role as the dominant downstream executor in this regulatory cascade.

To evaluate the clinical relevance of the METTL3‐IGF2BP2‐PUM2 axis, we analyzed protein expression in 12 paired OC tissues and normal fallopian tube epithelia tissues. Western blotting revealed significant upregulation of PUM2, METTL3, and IGF2BP2 in tumour tissues compared with normal controls (Figure 7G). We evaluated the correlation between PUM2 and METTL3 or IGF2BP2 with their corresponding mRNA levels by the TCGA‐OC cohort data. Pearson correlation analysis of TCGA data identified strong positive associations between PUM2 and METTL3, PUM2 and IGF2BP2 (Figure 7H). Moreover, by analyzing the TCGA dataset, we found that a high expression level of METTL3 mRNA predicted relatively poor PFS and OS in OC patients (Figure S6G). Consistently, higher expression of IGF2BP2 and PUM2 was associated with worse PFS and OS in OC patients (Figure S6H,I). RNA‐seq analysis of METTL3‐ and IGF2BP2‐knockdown OVCAR3 cells identified 2704 and 2505 differentially expressed genes (DEGs), respectively (Figure S6J; Tables S9 and S10). Subsequent intersection with PAS‐seq‐derived APA‐regulated genes revealed 84 co‐regulated transcripts exhibiting both expression changes and APA isoform switching (Figure S6K; Table S11). Functional annotation of these targets highlighted enrichment in vascular transport, membrane microdomain organization, and cadherin binding, along with activation of chronic myeloid leukaemia pathways, which could implicate METTL3‐IGF2BP2‐mediated APA regulation as a critical coordinator of membrane signalling in OC progression (Figure S6L). Overall, these results confirmed that PUM2 is an essential target of IGF2BP2 in OC pathogenesis. We revealed a novel mechanism of the m6A modification‐mediated APA process and identified the METTL3/IGF2BP2/PUM2 axis as a critical mediator of the initiation and progression of OC, which could provide an effective therapeutic and prognostic biomarker for OC patients.

In summary, our study revealed that METTL3/IGF2BP2 exerts an oncogenic effect on OC cells by promoting the 3′UTR shortening of PUM2, which harbours m6A modification and undergoes APA processing, in which the m6A reader IGF2BP2 directly interacts with the APA factor CPSF6 to regulate APA in an m6A‐dependent manner.

## 4 DISCUSSION

Among posttranscriptional modifications, m6A methylation is the most common modification of mRNAs and noncoding RNAs in eukaryotes and plays various important roles in many physiological and pathological processes.^3^ Dysregulation of m6A modification is involved in proliferation, apoptosis, migration, drug resistance, and cancer stem cell development in OC.^35^ Usually, m6A modification occurs with the consensus RRACH sequence (R = A, G; H = A, C, U), which is mainly enriched near stop codons, in 3′UTRs and the last exon in mRNA.^36^ Recently, APA has been shown to act as a general conserved mechanism of posttranscriptional regulation by which the pre‐mRNA 3′end is cleaved and polyadenylated to generate distinct transcript isoforms.^37^ Notably, most PASs are located in the 3′UTR near or partially overlapping with m6A sites, and m6A methylation could affect the choice of PAS in several diseases via APA factors.^38^ In fact, the m6A has been shown to regulate 3′‐end length through affecting APA processing.^21^ In the brain and liver tissues of mice, knocking down components of the methylase complex (METTL3, METTL14, and WTAP) resulted in increased proximal APA usage of most transcripts.^39^ Moreover, FTO knockout increased the number of distal APA sites in HEK293T cells.^40^ However, Molinie et al.^41^ reported a strong bias in that methylated transcripts tended to be linked to proximal APA sites, while nonmethylated transcripts tended to be linked to distal APA sites according to m6A‐LAIC‐seq in H1‐ESC and GM12878 cell lines. These findings prompted us to examine the interaction between m6A and APA in more detail. However, its regulatory mechanism awaits further research.

In our study, we observed a correlation between m6A methylation and APA processing in OC. The colocalization of m6A peaks and proximal PAS (pPAS) within 3′UTRs suggests a coordinated regulatory mechanism. By m6A‐seq, we revealed that m6A sites were highly enriched near APA events and that canonical APA motifs were enriched in OVCAR3 cells, suggesting that m6A sites are intimately associated with APA. Knockdown of METTL3 led to the shift towards distal PAS (dPAS) usage and 3′UTR lengthening in OVCAR3 cells, which supported a model wherein m6A deposition facilitates pPAS selection. Then, we tested the association of APA core factors with RNA oligonucleotides carrying m6A modifications. The results showed that only CPSF6 was more inclined to bind to RNA with m6A modification. While the m6A‐binding protein YTHDC1 has been shown to interact with APA factors FIP1L1 and CSTF3,^42^, ^43^ our findings indicated comparable FIP1L1/CSTF3 levels between ss‐A and ss‐m6A RNA samples. CPSF6 acts as an important regulator in APA processes by binding NUDT21 and composing CFIm subunits.^44^ Many studies have shown that CPSF6 is widely involved in the regulation of HIV‐1 infection via the immune system, but is independent of APA.^45^, ^46^ In cancers, CPSF6 plays an oncogenic role in hepatocellular carcinoma and gastric cancer by promoting the proliferation, migration, and invasion of cancer cells in vitro and in vivo via APA regulation.^47^, ^48^ Therefore, our preliminary exploration indicated that CPSF6 might be a key factor in the interaction between m6A modification and the APA process.

Previous studies have shown that m6A regulators can interact with APA core factors.^42^ The m6A writer VIRMA has been verified to be associated with CPSF6 in HeLa cells.^29^ Our IP results showed that VIRMA, MELLT3, and METTL14 interacted with CPSF6 in an RNA‐dependent manner in OC cells. CPSF6 is primarily localized within the nucleus, and m6A modification and APA processing are completed inside the nucleus, suggesting that m6A regulators and APA factors interact with each other within the nucleus. We detected the binding of conventional endonuclear m6A regulatory factors to CPSF6. However, no endonuclear m6A regulators were detected that directly bind to CPSF6 in OC cells, including YTHDC1, which has been reported previously in mouse oocytes.^49^ We further applied MS, co‐IP, and immunofluorescence assays to screen and verify the CPSF6‐binding proteins. Surprisingly, the IGF2BP1/2/3 complex substantially colocalized with CPSF6 and directly bound to CPSF6 in A2780 and OVCAR3 cells. Insulin‐like growth factor‐2 (IGF2) mRNA‐binding proteins 1, 2, and 3 (IGF2BP1/2/3) are newly identified m6A reader proteins that regulate mRNA stability and translation in the cytoplasm by recognizing m6A sites.^25^ Thus, we speculate that the IGF2BP1/2/3 complex recognizes m6A modifications and recruits CPSF6 to participate in APA regulation. It was previously reported that the CPSF6 protein contains three distinct domains: an N‐terminal RNA recognition motif (RRM), a midterm proline‐rich region (PRR) and a C‐terminal arginine/serine‐rich (RS) domain.^50^, ^51^ The RRM domain can recognize specific RNA sequences and bind to the NUDT21 and NXF1 proteins.^33^, ^52^ The PRR domain is an intrinsically disordered region (IDR) that is associated with viral infections by binding to the HIV‐1 capsid protein p24.^53^ The RS domain is usually phosphorylated and involved in the regulation of alternative splicing by interacting with other RS domain proteins, such as SRSF3, SRSF7 or SRSF10.^31^, ^50^ In our research, we revealed that CPSF6 directly bound to the IGF2BP1/2/3 complex through the RS domain. Interestingly, the CPSF6 RS domain, instead of the RRM domain, also shows RNA binding activity.^31^ Additionally, Zhu et al.^54^ noted that the RS domains of CPSF6 could directly interact with FIP1.

To determine how the CPSF6 RS domain interacts with the IGF2BP1/2/3 complex, the CPSF6 RS domain, IGF2BP1, IGF2BP2 and IGF2BP3 proteins were individually expressed and purified. GST pulldown assays revealed that IGF2BP2 directly bound to the CPSF6 RS domain in vitro. IGF2BP2 may be a crucial m6A factor for regulating the APA process. Furthermore, we observed that the C‐terminal KH domain of IGF2BP2 directly bound to CPSF6. IGF2BP2 is composed of six RNA‐binding motifs: two RRM domains at the N‐terminus and four KH domains at the C‐terminus. The KH domains are indispensable for binding a specific m6A recognition element, whereas the RRM domains mediate protein‒RNA complex stabilization and interactions with other RBPs.^25^ Therefore, we propose that IGF2BP2 acts as a regulator of APA processing, likely by directly recognizing the m6A modification and binding to the CPSF6 RS domain through the KH domains.

We observed that knocking down METTL3 and IGF2BP2 significantly promoted the use of dPASs of target genes, indicating that m6A modification may increase the use of pPASs in OC cells, leading to a shortening in the 3′UTR of genes. Furthermore, we screened potential target genes harbouring m6A sites bound to both IGF2BP2 and CPSF6, which resulted in concurrent m6A methylation and APA. After multiple experiments, we confirmed that the 3′UTR of PUM2 was lengthened when METTL3 and IGF2BP2 were knocked down in OC cells. PUM2 was verified to be an m6A transcript that bound to IGF2BP2 and CPSF6. The regions of m6A sites and CPSF6 binding sites are located in the same region of the PUM2 3′UTR. The mutation of m6A sites in PUM2 3′UTR could affect the expression of the PUM2 short transcript.

Pumpilio RNA‐binding family member 2 (Pumilio2, PUM2) is an RBP that affects multiple aspects of mRNA metabolism and posttranscriptional regulation.^55^ PUM2 is involved in multiple cellular responses and disease processes, including cancer development and progression.^56^, ^57^ However, the function of PUM2 in OC has not yet been described. We found that knockdown of METTL3 and IGF2BP2 reduced the protein expression of PUM2. Similarly, METTL3 inhibitor STM2457 and IGF2BP2 inhibitor CWI1‐2 decreased the protein expression of PUM2. Conversely, knockdown of FTO increased the protein expression of PUM2. And knockdown of YTHDF1 and YTHDF2 had no effect on PUM2 protein expression. Since METTL3 and IGF2BP2 are essential for mRNA stability,^58^, ^59^ we found that METTL3 and IGF2BP2 knockdown reduced the stability of PUM2 mRNA in OC cells via a half‐life experiment. The 3′UTR of PUM2 has previously been found to interact with miRNAs.^60^ We speculated that the resultant shortened 3′UTR of PUM2 likely promoted oncogenesis by eliminating miRNA‐binding sites while preserving essential cis‐elements for RNA stability. Thus, our results suggest that METTL3 and IGF2BP2 are useful for regulating APA processing of PUM2 3′UTR, which contains m6A sites and CPSF6 binding sites, to enhance RNA stability.

Although the functions of many m6A regulators, including IGF2BP2, have been reported in OC,^61^ we again confirmed that IGF2BP2 acted as an oncogene in OC and that downregulated IGF2BP2 expression inhibited OC proliferation and metastasis in vitro and in vivo. On the other hand, we found PUM2 acted as an oncogene to promote the proliferation and metastasis of OC cells. And, the rescue assays showed that overexpression of PUM2 could reverse IGF2BP2 inhibition‐driven suppression of malignant phenotypes, and PUM2 knockdown could attenuate the IGF2BP2 protumorigenic effect. Moreover, we found a significant correlation between PUM2 and METTL3/IGF2BP2 expression in OC patients, and high expression of METTL3/IGF2BP2/PUM2 was associated with poor prognosis in patients with OC. While this study establishes METTL3/IGF2BP2‐mediated m6A modification as a critical regulator of APA dynamics in OC, certain limitations inherent to the experimental approach merit acknowledgement. The reliance on subcutaneous xenograft models, though practical for preliminary therapeutic assessments, may incompletely model the immunosuppressive tumour microenvironment (TME) or patient‐derived xenografts (PDXs). Furthermore, we just identified IGF2BP2‐CPSF6 as APA regulators; the precise mechanisms and the global changes linking m6A deposition to APA processing remain unresolved.

In conclusion, our study revealed that IGF2BP2 directly interacts with CPSF6, mediating the APA regulation and recognizing the m6A site of PUM2, thereby increasing the usage of pPAS and promoting the generation of short PUM2 transcripts with greater mRNA stability. This is an unrecognized mechanism by which IGF2BP2 inhibits tumour progression in an m6A cooperated with APA manner. These findings provide a potential prognostic marker for OC and suggest that targeting the METTL3‐IGF2BP2/CPSF6‐PUM2 axis activity is a promising strategy for treating patients with OC.

## AUTHOR CONTRIBUTIONS

Yong Zhu, Ping Yi, and Jing Xu conceived and designed the study. Xin Luo, Qinglv Wei, and Lingcui Xie performed experiments and analyzed the data. Ningxuan Chen, Jiani Xu, Xiaoyan Jiang, and Xinzhao Zuo carried out xenograft experiments. Bin Gu provided the development of the methodology. Hongyan Zhao, Xiaoyi Liu, Yu Yang, and Tao Liu provided assistance in cell and biochemistry experiments. Xin Luo prepared the figures and wrote the original manuscript. Yong Zhu, Ping Yi, and Jing Xu revised the manuscript. Tao Liu, Yong Zhu, Ping Yi, and Jing Xu provided constructive suggestions and help for this paper. Ping Yi and Jing Xu supervised the project and provided the funding support.

## CONFLICT OF INTEREST STATEMENT

The authors declare no conflict of interest.

## ETHICS STATEMENT

All human tissue studies were conducted in accordance with the Declaration of Helsinki and approved by the Institutional Review Board of the Third Affiliated Hospital of Chongqing Medical University (approval no. 202293). All animal studies were conducted in accordance with the Institutional Animal Care and Use Committee of Chongqing Medical University (approval number: IACUC‐CQMU‐2023‐0153).

## Supporting information



Supporting Information

Supporting Information

## Data Availability

The data that support the findings of this study are available from the corresponding author upon reasonable request. Additional materials and methods are described in the Supporting Information Data.
