# Supplementary material for: IGF2BP2 binding to CPSF6 facilitates m6A‐mediated alternative polyadenylation of PUM2 and promotes malignant progression in ovarian cancer
Source: Clin Transl Med. 2025 Jul 9;15(7):e70388. doi: 10.1002/ctm2.70388 (PMC12238680; doi:10.1002/ctm2.70388)
Supplement: Supplementary file 1 — Supporting Information [file CTM2-15-e70388-s002.docx]

**Supplementary information**

**IGF2BP2 binding to CPSF6 facilitates m6A mediated alternative polyadenylation of PUM2 and promotes malignant progression in ovarian cancer**

Xin Luo^1,2^, Qinglv Wei^3^, Lingcui Xie^1,2^, Ningxuan Chen^1,2^, Bin Gu^4^, Jiani Xu^1,2^, Xiaoyan Jiang^1,2^, Xinzhao Zuo^1,2^, Hongyan Zhao^5^, Xiaoyi Liu^1,2^, Yu Yang^1,2^, Tao Liu^1,2^, Yong Zhu^4^, Ping Yi^1,2^, Jing Xu^1,2^

^1^Department of Obstetrics and Gynecology, The Third Affiliated Hospital of Chongqing Medical University, Chongqing, P. R. China.

^2^Chongqing MunicipalHealth Commission Key Laboratory of Basic and Clinical Transformation of Gynecological Oncology (The Third Affiliated Hospital of Chongqing Medical University)

^3^Chongqing Key Laboratory of Child Infection and Immunity, Children’s Hospital of Chongqing Medical University, Chongqing, P. R. China.

^4^School of Basic Medicine, Chongqing Medical University, Chongqing, P. R. China.

^5^School of Basic Medicine, Hubei University of Medicine, Shiyan, Hubei, P. R. China.

**Supplemental Figures S1-S6**

**Supplemental Tables S1**

**Supplementary figures**


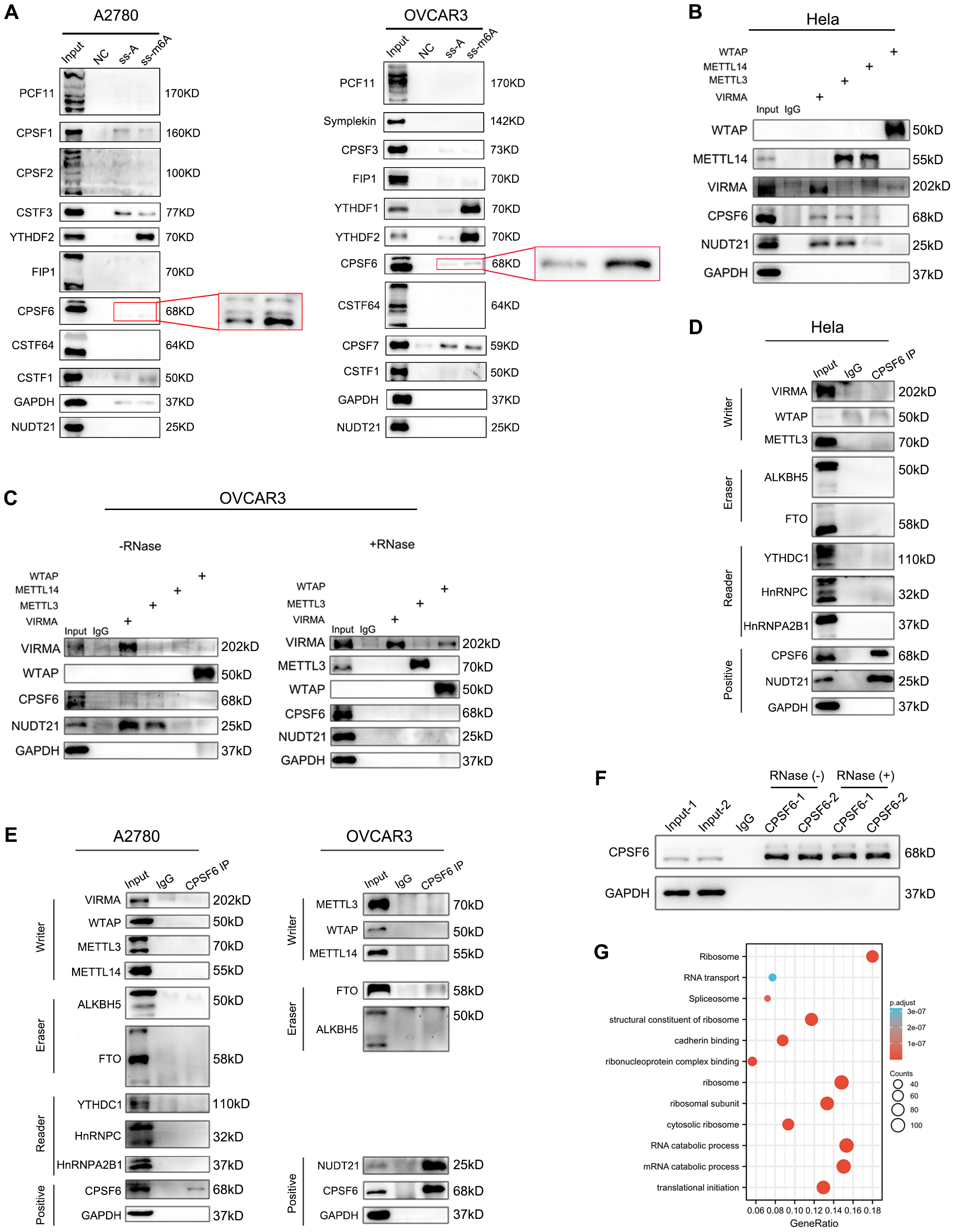


**Figure S1. Screening of APA core regulators and relevant m6A regulators in OC cells.** **(A)** Screening of APA core regulators related to m6A modification by RNA‒protein pulldown assay. **(B)** Cell lysate of HeLa cells was immunoprecipitated with m6A "writer" VIRMA, METTL3, METTL14 and WTAP to detect APA regulators CPSF6 and NUDT21. **(C)** Cell lysate of OVCAR3 cells was immunoprecipitated with VIRMA, METTL3, METTL14 and WTAP with or without RNaseA to detect CPSF6 and NUDT21. **(D)** IP assay with CPSF6 to detect m6A regulators in HeLa cells. **(E)** Cell lysates of A2780 and OVCAR3 cells were immunoprecipitated with CPSF6 to detect m6A regulators. **(F)** Quality control of CPSF6 IP/MS with or without RNaseA. **(G)** GO enrichment analysis of CPSF6 IP proteins.


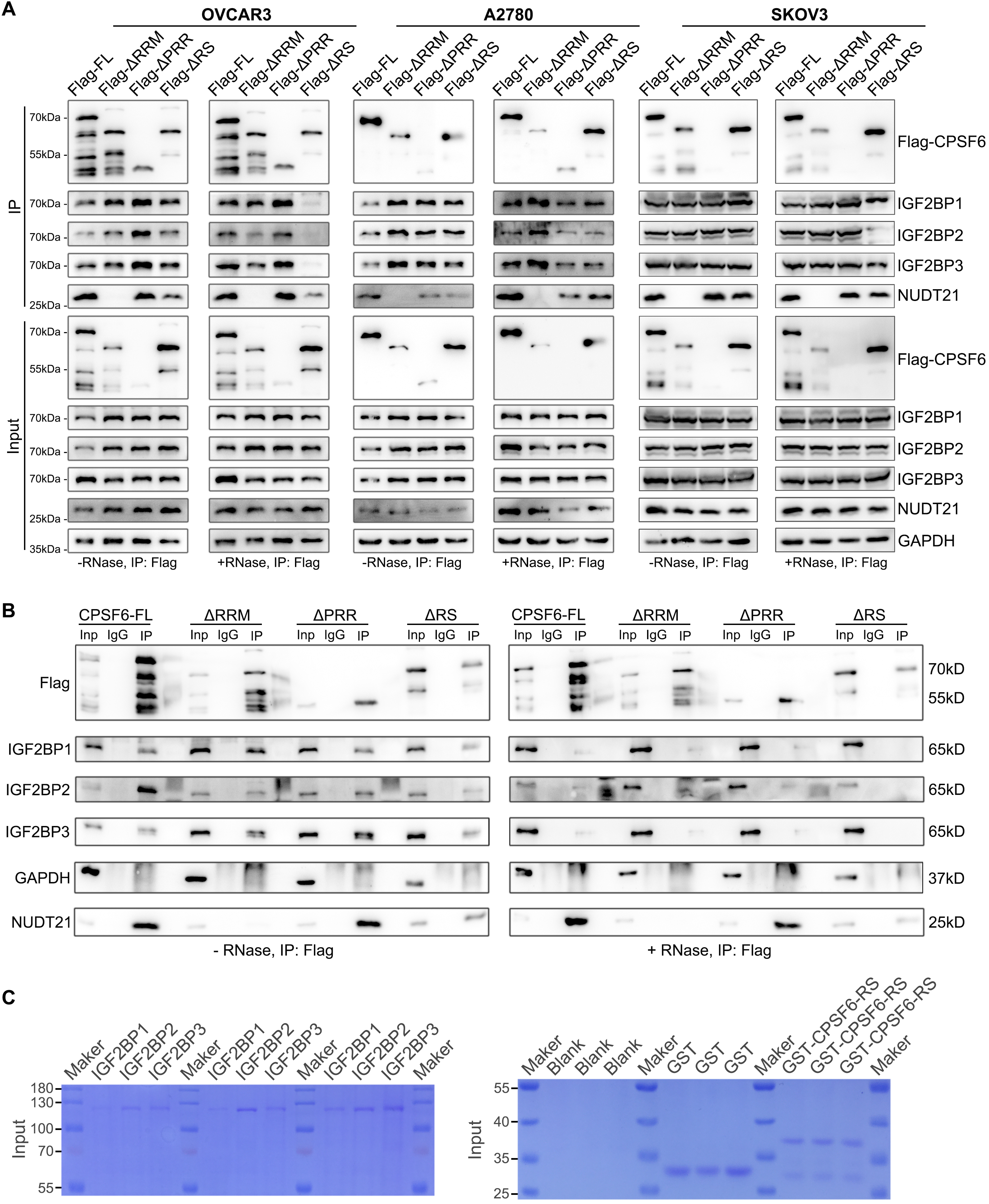


**Figure S2. Binding of CPSF6 truncation to IGF2BP1/2/3 protein in OC cells. (A)** IP assay of CPSF6 truncated protein in OC cells. **(B)** IP assay of CPSF6 truncated protein in OVCAR3 cells with or without RNase. **(C)** Coomassie brilliant blue staining of GST, GST-CPFS6-RS and His-IGF2BP1/2/3 proteins.


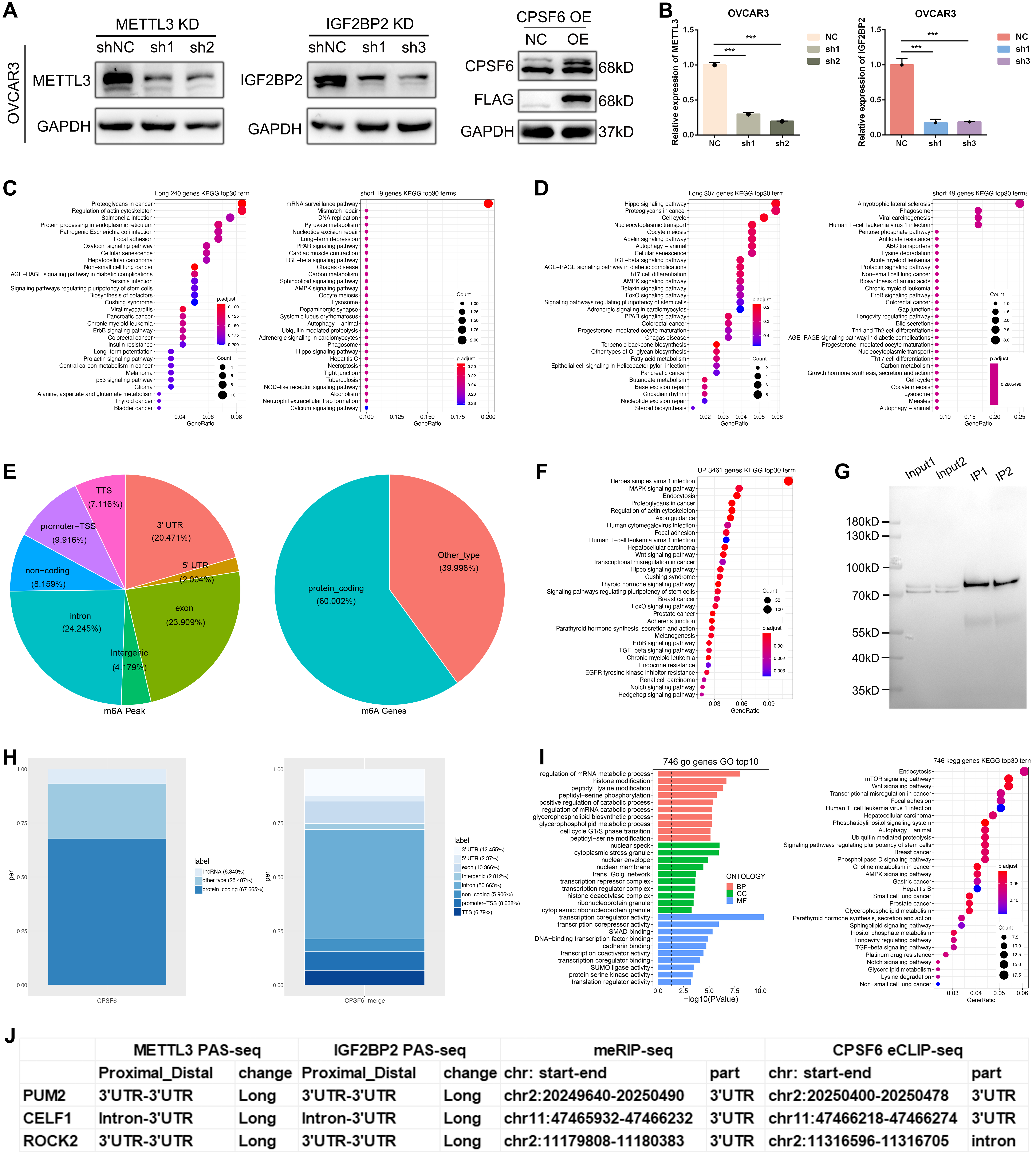


**Figure S3. Screening of target genes for m6A and APA regulation using OVCAR3 cells.** **(A)** Detection of knockdown and overexpression efficiency by Western blot. **(B)** Detection of the knockdown efficiency by RT‒qPCR. **(C)** KEGG enrichment analysis of APA genes after METTL3 knockdown. **(D)** KEGG enrichment analysis of APA genes after IGF2BP2 knockdown. **(E)** The binding region of m6A peak and the type of RNA were identified by meRIP-seq. **(F)** KEGG enrichment of IGF2BP2 binding genes. **(G)** Quality control of CPSF6 eCLIP-seq assay. **(H)** The distribution of target gene types and binding regions determined by eCLIP-seq. **(I)** GO function and KEGG pathway enrichment analysis of target genes by CPSF6 eCLIP-seq. **(J)** The list of target genes.

**
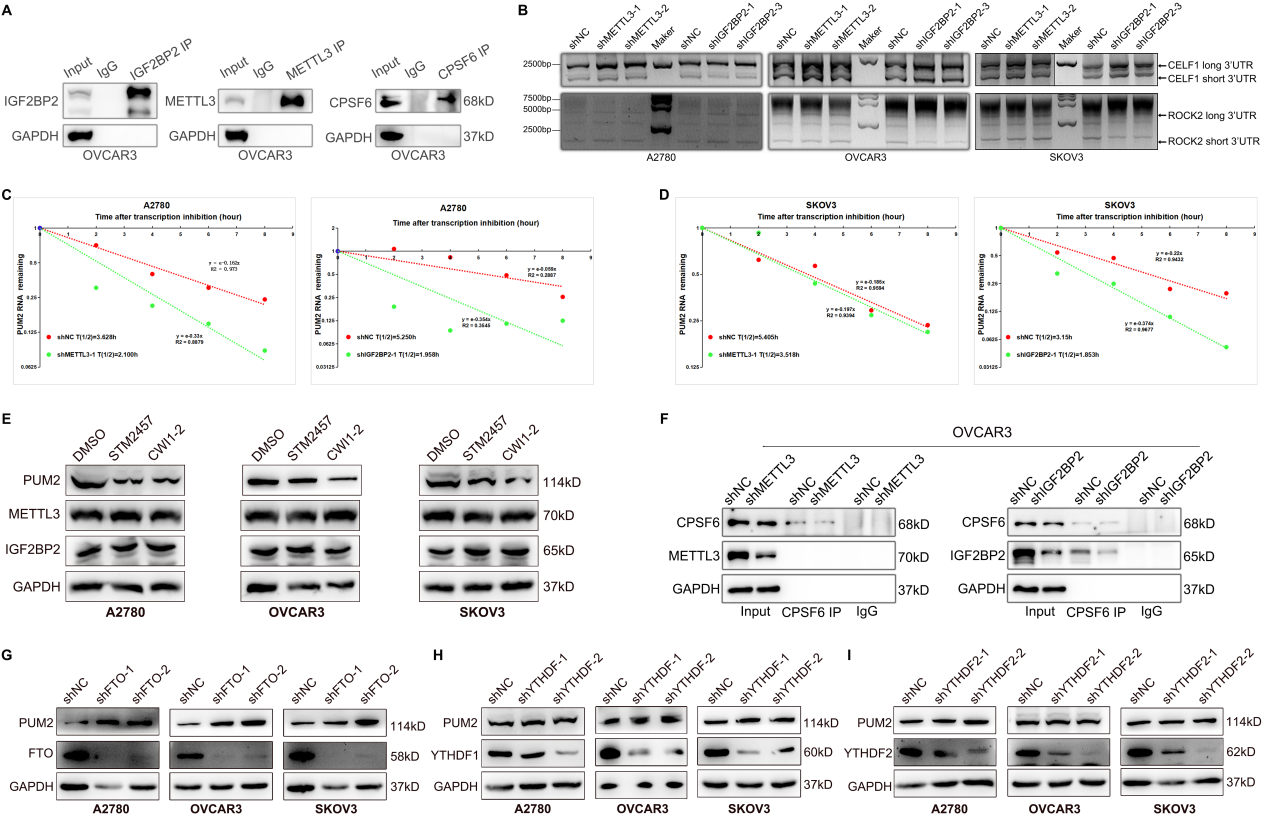
Figure S4. METTL3 and IGF2BP2 regulate the APA process and mRNA stability of PUM2 by recruiting CPSF6.** **(A)** Quality control of METTL3 and IGF2BP2 RIP-qPCR as well as CPSF6 eCLIP-qPCR using western blotting. **(B)** The abundance of CELF1 and ROCK2 transcripts of different lengths in OC cells with METTL3 or IGF2BP2 knockdown was determined by 3’ RACE. **(C and D)** Decay curves of PUM2 mRNA after METTL3 and IGF2BP2 knockdown in A2780 **(C)** and SKOV3 (D) cells. **(E)** The expression of PUM2 was detected by western blot after inhibiting by STM2457 and CWI1-2 treatment. **(F)** Co-IP analysis of endogenous CPSF6 in OVCAR3 cells in which METTL3 or IGF2BP2 expression was knocked down for RIP-qPCR assay. **(G-I)** The expression of PUM2 was detected by western blot after knocking down FTO **(G)**, YTHDF1 **(H)** and YTHDF2 **(I)** in OC cells.


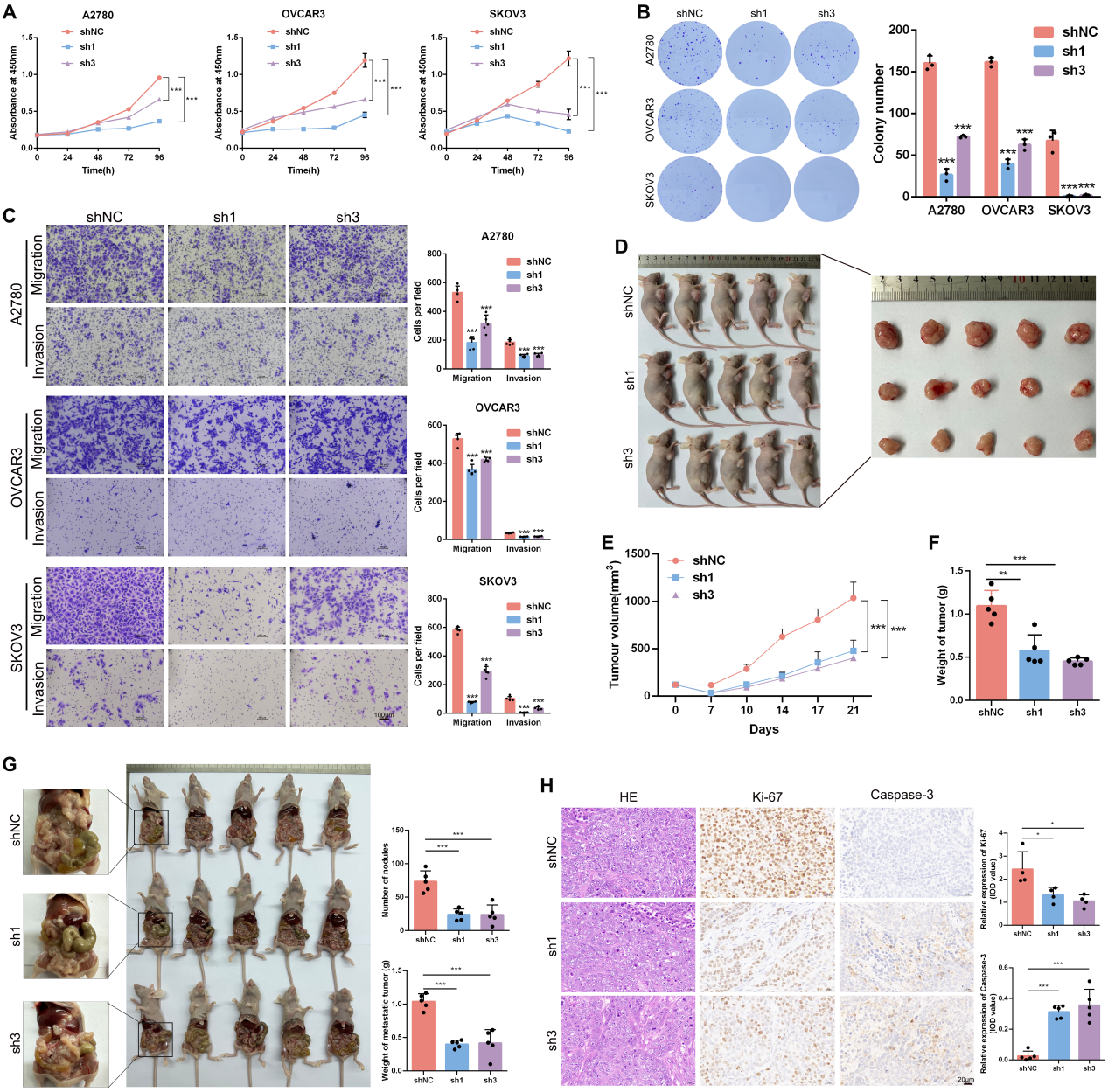


**Figure S5. Downregulation of IGF2BP2 expression inhibited the growth and metastasis of OC cells *in vitro* and *in vivo*.** **(A-C)** CCK-8 **(A)**, colony formation **(B)** and Transwell **(C)** assays of OC cells infected with pLKO.1-shIGF2BP2 or pLKO.1-control. **(D-F)** Gross xenograft model anatomy **(D)** growth curve **(E)** and tumor weight **(F)** after subcutaneous injection of IGF2BP2-knockdown OVCAR3 cells into nude mice. **(G)** To establish an intraperitoneal metastasis model, OVCAR3 cells with IGF2BP2 knockdown were injected intraperitoneally into nude mice, and the graphs show the gross anatomy of the tumors, number of metastatic nodules and metastatic tumor weight data. **(H)** HE staining and Ki-67 and Caspase-3 IHC staining of subcutaneous tumors. **P* < 0.05, ***P* < 0.01, ****P* < 0.001.


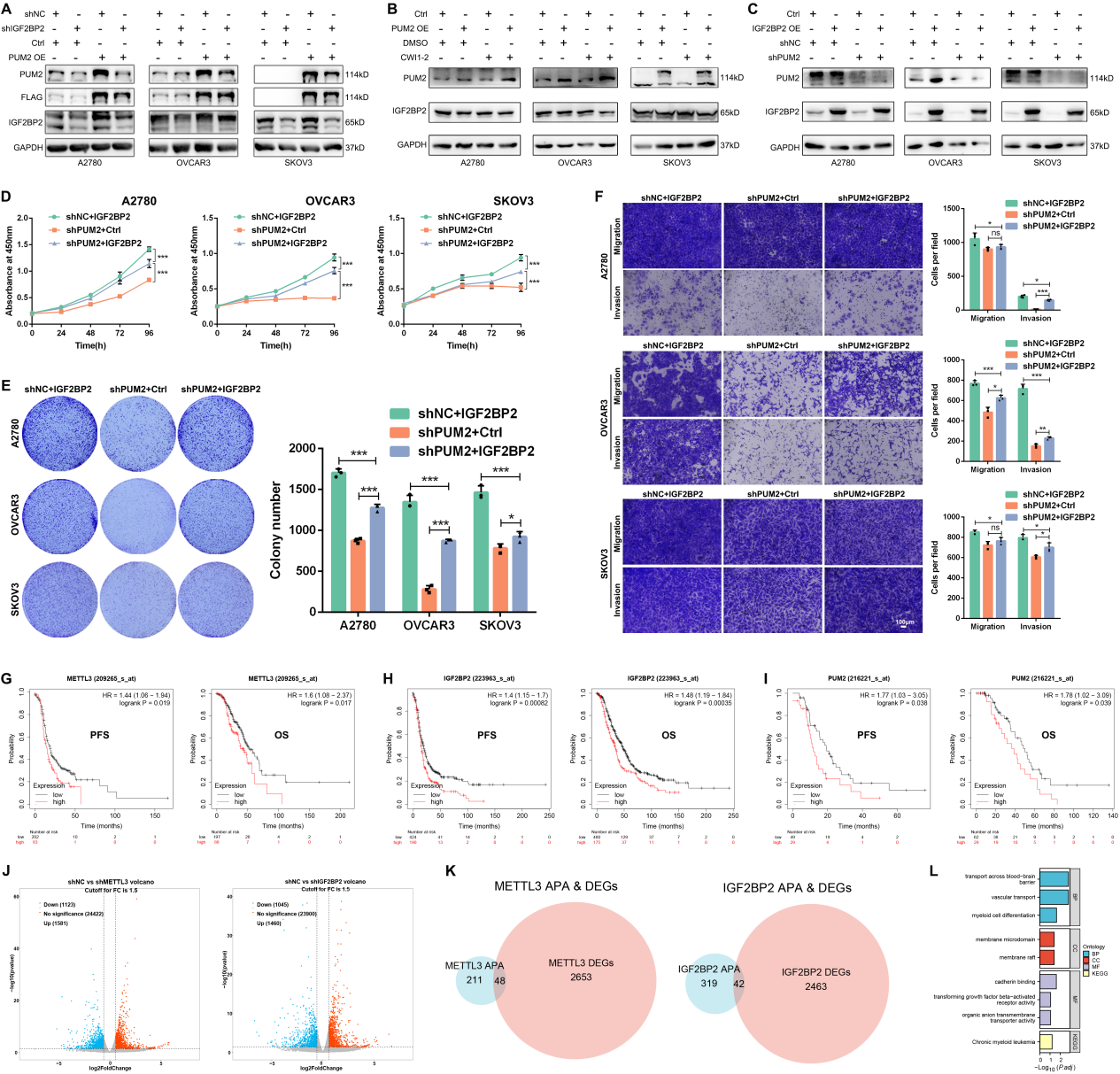


**Figure S6. METTL3-IGF2BP2-PUM2 axis promoted OC progression and increased poor prognosis with patients.** **(A)** Detection of protein expression in OC cells in which IGF2BP2 was knocked down and PUM2 was overexpressed. **(B)** The protein expression of OC cells was detected after CWI1-2 treatment and PUM2 overexpression. **(C)** Detection of protein expression in OC cells in which PUM2 was knocked down and IGF2BP2 was overexpressed. **(D-F)** Knockdown of PUM2 reversed the growth **(D)**, proliferation **(E)** and metastasis **(F)** of OC cells in which IGF2BP2 was overexpressed. **(G-I)** Kaplan–Meier analysis of the PFS and OS rates associated with METTL3 **(G)**, IGF2BP2 **(H)** and PUM2 **(I)** in OC patients from the TCGA cohort. **(J and K)** Differential expression gene (DEG) detection **(J)** and APA conjoint analysis **(K)** using RNA seq with METTL3 and IGF2BP2 knockdown in OVCAR3 cells. **(L)** The GO and KEGG term analysis of APA & DEGs.

**Supplementary tables**

**Table S1. Sequences of primers used in this study**

| **Name** | **Sequence** (5’–3’) |
| --- | --- |
| shRNA target oligo | |
| shMETTL3-1 | GCCTTAACATTGCCCACTGAT |
| shMETTL3-2 | GCAAGTATGTTCACTATGAAA |
| shFTO-1 | TCACGAATTGCCCGAACATTA |
| shFTO-2 | CCCATTAGGTGCCCATATTTA |
| shIGF2BP2-1 | AGTGAAGCTGGAAGCGCATAT |
| shIGF2BP2-3 | TTCCCGCATCATCACTCTTAT |
| shPUM2-1 | CCTAATCCTACAGCTAATAAA |
| shPUM2-2 | CTAGCTCCAACTGCCTATTAT |
| shYTHDF1-1 | GATACAGTTCATGACAATGA |
| shYTHDF1-2 | GAAACGTCCAGCCTAATTCT |
| shYTHDF2-1 | GATGGATTAAACGATGATGAT |
| shYTHDF2-2 | GCAGACTTGCAGTTTAAGTAT |
| Primers used in PCR | |
| METTL3-F | TTGTCTCCAACCTTCCGTAGT |
| METTL3-R | CCAGATCAGAGAGGTGGTGTAG |
| GAPDH-F | GTCAAGGCTGAGAACGGGAA |
| GAPDH-R | AAATGAGCCCCAGCCTTCTC |
| IGF2BP2-F | AGTGGAATTGCATGGGAAAATCA |
| IGF2BP2-R | CAACGGCGGTTTCTGTGTC |
| PUM2-F | GTAGTGATGCTTTCCTTGGTTGT |
| PUM2-R | CAAAGAGGGCAGGGACGTAAA |
| ROCK2-F | AGGTGATCGTATTCTTCCAGTG |
| ROCK2-R | TTACAGGGAAAAGGGGAACACA |
| CELF1-F | TGGAGACAAACCTGTGTGAG |
| CELF1-R | TTGAATCCCGTTGTTGCTGC |
| ROCK2-Pro-F | GCCTTCTATGAAAGCAGTCATTAT |
| ROCK2-Pro-R | GGGCCATCATATTTCAGTCTTGT |
| ROCK2-Dis-F | CCTCTCCCTGACTCCCATC |
| ROCK2-Dis-R | ACATTACTACGAAGATGCAACAA |
| CELF1-Pro-F | CCCCTCTGAGACTGGAGTGA |
| CELF1-Pro-R | AGCGAAACTCCCACAGAAGG |
| CELF1-Dis-F | TTGGAGCAAGGGAGTATGTGTT |
| CELF1-Dis-R | AAGGAAGGTACCACACTTGGC |
| PUM2-Pro-F | ATATTCAGCCCTGAGTGGAGAC |
| PUM2-Pro-R | ACATCATATACAGGCATTCTGTGC |
| PUM2-Dis-F | GTCCTGGCTCTTATTGGTTCA |
| PUM2-Dis-R | TGAGGTACAGAGGGGTATTTT |
| Primers used in 3'RACE | |
| PUM2-RACE | GATTACGCCAAGCTTCAGCCCTGAGTGGAGACCTATCAGATTG |
| ROCK2-RACE | GATTACGCCAAGCTTCTAACTGCCTTCTATGAAAGCAGTCATT |
| CELF1-RACE | GATTACGCCAAGCTTCTCTGAGGCTCCTGAGCACATTTTGC |
